# Phosphate promotes Arabidopsis root skewing and circumnutation through reorganisation of the microtubule cytoskeleton

**DOI:** 10.1111/nph.20152

**Published:** 2024-10-03

**Authors:** Hui Sheng, Harro J. Bouwmeester, Teun Munnik

**Affiliations:** ^1^ Plant Cell Biology, Green Life Sciences Cluster, Swammerdam Institute for Life Sciences University of Amsterdam Science Park 904 Amsterdam 1098 XH the Netherlands; ^2^ Plant Hormone Biology Group, Green Life Sciences Cluster, Swammerdam Institute for Life Sciences University of Amsterdam Science Park 904 Amsterdam 1098 XH the Netherlands

**Keywords:** Arabidopsis, circumnutation, microtubule cytoskeleton, phosphate, root skewing

## Abstract

- Phosphate (P_i_) plays a key role in plant growth and development. Hence, plants display a range of adaptations to acquire it, including changes in root system architecture (RSA). Whether P_i_ triggers directional root growth is unknown.
- We investigated whether Arabidopsis roots sense P_i_ and grow towards it, that is whether they exhibit phosphotropism. While roots did exhibit a clear P_i_‐specific directional growth response, it was, however, always to the left, independent of the direction of the P_i_ gradient.
- We discovered that increasing concentrations of KH_2_PO_4_, trigger a dose‐dependent skewing response, in both primary and lateral roots. This phenomenon is P_i_‐specific – other nutrients do not trigger this – and involves the reorganisation of the microtubule cytoskeleton in epidermal cells of the root elongation zone. Higher P_i_ levels promote left‐handed cell file rotation that results in right‐handed, clockwise, root growth and leftward skewing as a result of the helical movement of roots (circumnutation).
- Our results shed new light on the role of P_i_ in root growth, and may provide novel insights for crop breeding to optimise RSA and P‐use efficiency.

## Introduction

Phosphorus (P) is an essential macronutrient for the growth and development of every living organism, including plants. It is a component of many metabolites and cellular building blocks, including nucleic acids, ATP, and phospholipids, and plays key roles in the plant's energy balance, carbon assimilation, signal transduction, and many other processes (Vance *et al*., 2003; Jain *et al*., 2007; Rouached *et al*., 2010). Plants absorb P in the form of inorganic phosphate (P_i_), which is taken up from the soil through their root system (Schachtman *et al*., 1998; Cho *et al*., 2021). The concentration of P_i_ in the soil is often limiting and usually not homogeneously distributed, mainly residing in the top soil due to its strong negative charge (Chu & Chang, 1966; Ahn & Keter, 1986; Pothuluri *et al*., 1986; Péret *et al*., 2011; Motte *et al*., 2019; Huang & Zhang, 2020). Availability is further affected by pH, microbial competition, and by the presence of various cations (Fujii *et al*., 2005; Richardson *et al*., 2009; Plassard *et al*., 2019).

To increase the uptake of P_i_ and to regulate its internal utilisation, plants have developed a variety of systemic responses when P_i_ is limiting, which is regulated by a complex signalling network of genes, hormones, microRNAs (miRNA) and other signalling molecules (Franco‐zorrilla *et al*., 2004; Gutiérrez‐Alanís *et al*., 2018; He *et al*., 2023). Typically, phosphate starvation‐induced (*PSI*) genes are upregulated by P_i_ deficiency, which are involved in the acquisition and remobilisation of P_i_ to maintain its P homeostasis, and are often used as markers for the P_i_ Starvation Response (PSR) (Chiou & Lin, 2011; Wang *et al*., 2018; Fabia *et al*., 2019). Changes in carbon metabolism, membrane composition and root system architecture (RSA) are hallmarks of this PSR (Castrillo *et al*., 2017; Crombez *et al*., 2019; Xu *et al*., 2020; Wang *et al*., 2021). Inhibition of primary root growth and promotion of lateral root growth and root hair proliferation to promote P_i_ absorption are hallmarks of such RSA changes (Rouached *et al*., 2010; Péret *et al*., 2011; Dong *et al*., 2017).

The plants's RSA is a net result of various environmental factors, including gravity, water, light, salinity, nutrition, and touch, making it highly plastic in its development (Gilroy & Masson, 2008; Galvan‐Ampudia *et al*., 2013; Eysholdt‐Derzsó & Sauter, 2017; Karban, 2021). Any directional growth change in response to such stimuli is called a tropism, and those for gravitropism, hydrotropism, phototropism, halotropism, chemotropism, nutritropism, and thigmotropism, are well established, and ensure that plants optimise their growth in response to environmental signals (Galvan‐Ampudia *et al*., 2013; Su *et al*., 2017; Yamazaki *et al*., 2020; Kawamoto & Morita, 2022; Li *et al*., 2022; Yu *et al*., 2022).

With P_i_ being such a crucial nutrient, we wondered whether roots are able to sense P_i_ and whether they use such information to direct their growth towards P_i_‐containing patches (i.e. phosphotropism). Already in 1904, directional growth towards P_i_ was reported in roots of white lupine, which at the time was referred to as chemotaxis (Newcombe & Rhodes, 1904). Here, we show that Arabidopsis grown on agar plates do not exhibit phosphotropism. Nonetheless, we found that P_i_ has a dramatic effect on the circumnutation of roots, which ultimately affects the behaviour of both primary‐ and lateral roots, generating a RSA that could be beneficial for prolonged uptake of P_i_.

## Materials and Methods

### Plant material and growth conditions

*Arabidopsis thaliana*, ecotypes Columbia (Col‐0) and Wassilewskija (Ws‐4) were used as reference lines in this study. Seeds were surface sterilised using 1 ml of 50% bleach and 60 μl of 70% ethanol for 10 min and then washed five times with sterilised water.

Seeds were sown on square petri dishes (12 × 12 cm) containing 40 ml of ½‐strength Murashige & Skoog medium (½MS) (Murashige & Skoog, 1962), supplemented with 1% (w/v) sucrose, 1% (w/v) Daishin agar (Duchefa), and set to pH 5.8 (KOH). To vary the P_i_ concentration, ½MS medium ‘without’ phosphate (Duchefa) was used, which was supplemented with different concentrations of P_i_, supplied from a 100 mM KH_2_PO_4_ stock solution. Differences in K^+^ were compensated using KCl. Plates were stratified for 2 d in the dark at 4°C, and placed vertically at a 70° angle in a growth chamber at 22°C with long‐day photoperiod (16 h : 8 h, light : dark) (see Supporting Information Fig. S1A).

To create a P_i_‐concentration gradient in agar plates, 40 μl of 100 mM phosphate buffer (K_2_HPO_4_/KH_2_PO_4_ mixture with pH 5.8) was pipetted onto a small piece of triangular filter paper (2 × 2 cm), which after drying was placed at the right‐ or left‐bottom corner of an ½MS plate containing 15 μM P_i_. As a control, 40 μl of MQ water was pipetted onto filter paper and placed, after drying, at the bottom corner.

### P_i_‐gradient visualisation

To visualise the diffusion of the phosphate from the filter paper into the agar, 40 μl of 1 M K_2_HPO_4_/KH_2_PO_4_ mixture (pH 5.8), containing a trace amount of radioactive ^32^P_i_ (1 μl of 50 nCi/mL ^32^P_i_ per 500 μl) was pipetted on a piece of filter paper, which after drying was placed in the corner of a low‐P_i_ (15 μM KH_2_PO_4_) medium agar plate. Plates were exposed to a PhosphoImager screen in the dark and were scanned after 1, 22, 50, 73, 97 and 121 hrs to visualise the development of the P_i_ gradient (Fig. S2).

### Quantification of root skewing

Roots were scanned from the back of the plates using an Epson Perfection V800 Photo Scanner (J221B). ImageJ was used to analyse root length (*L*), angle of deviation from the gravity vector of the root tip (θ), and length of the idealised root response (*L*_c_). *L*_*x*_ and *L*_*y*_ were obtained from Excel calculations (*L*_*x*_ = *L*_c_ × sin θ; *L*_*y*_ = *L*_c_ × cos θ) and the Horizontal Growth Index (HGI) was calculated (*L*_*x*_/*L*) as a measure of root skewing (Fig. S1B,C) (Grabov *et al*., 2005; Vaughn & Masson, 2011). For root pictures, images were inverted to give the front view orientation and the real direction of skewing.

### Epidermal cell file rotation and propidium iodide staining

Microscopic root images were routinely obtained using a Leica stereomicroscope (Leica MZFLIII, Leica Microsystems GmbH, Wetzlar, Germany) at ×5 magnification. To stain the cell walls, seedlings were incubated in dilute propidium iodide (10 μg ml^−1^; Sigma‐Aldrich, 81 845) for 3–5 min and washed twice in 6‐well plates filled with working buffer (2.5 mM MES and 1 mM KCl, pH 5.8) for 30 s. Seedlings were mounted on microscopic slides in working buffer and imaged using a Zeiss LSM510 confocal microscope (A Plan‐Apochromat ×20 objective) with an excitation at 488 or 514 nm. ImageJ was used to analyse the epidermal cell file angle deviation from the root growth direction.

### Microtubule drugs assays

Seedlings were grown on ½MS medium containing three different P_i_ concentrations (300, 625, 1250 μM) with or without 1 μM taxol (Paclitaxel, Cat# T7191; Sigma‐Aldrich) (from 10 mM Taxol stock in DMSO) or 3 μM propyzamide (Cat# 45645; Sigma‐Aldrich) (from 20 mM Propyzamide stock in DMSO). As control, the same amount of DMSO was added without the inhibitor. Seedlings were grown for 7 d and individual roots were imaged with a Leica stereomicroscope, or stained with propidium iodide and imaged using a Zeiss LSM510 confocal microscope as described above.

### Microtubule orientation analysis

Various *A. thaliana* microtubule reporter lines were used to image the microtubule dynamics of seedlings grown at 300, 625, or 1250 μM P_i_ for 6 d. *UBQ::GFP‐TUB6* (Col‐0 background) (Le & Ambrose, 2018) and *UBQ1::GFP‐MBD* (Col‐0) (Le & Ambrose, 2018) were kindly provided by Dr Chris Ambrose (Saskatoon, Canada); *TUA6‐GFP* (Col‐0) (Shaw *et al*., 2003; Vavrdová *et al*., 2020), *MAP4‐GFP* (Col‐0) (Marc *et al*., 1998; Komis *et al*., 2014; Tichá *et al*., 2020) and *pUBQ1::mRFP‐TUB6* (Col‐0) (Ambrose *et al*., 2011; Novák *et al*., 2018) were kindly provided by Dr Jozef Šamaj (Olomouc, Czech Republic); *UBQ10::EYFP‐TUB6* (Col‐0) (Sugiyama *et al*., 2017; Gunji *et al*., 2020) was kindly provided by Dr Joop Vermeer (Neuchâtel, Switzerland); *35S::GFP‐MBD* (Ws‐4 background) (Müller *et al*., 2007; Hamant *et al*., 2008; Hervieux *et al*., 2016) was kindly provided by Dr Olivier Hamant (Lyon, France). The microtubule cytoskeleton was analysed using a Zeiss LSM510 confocal microscope with a ×40 water objective (numerical aperture 1.2; excitation 488 nm, emission 516 nm). A region of interest (ROI) per cell was selected to quantify the orientation of the cortical microtubules using the OrientationJ plugin in ImageJ (Fonck *et al*., 2009; Rezakhaniha *et al*., 2012; Püspöki *et al*., 2016).

### Time‐lapse imaging and analysis of root dynamics

To acquire time‐lapse series of growing Arabidopsis seedlings on agar plates, a home‐made imaging system was constructed using an iPhone SE with a macro lens (*f* = 60 mm, ×0.75 magnification), which was positioned in front of the agar plate at an angle of 70°, the same as the plate itself (Fig. S1D). Images were captured every 10 min over a period of 3–6 d, using the OSnap application of iPhone. Primary root length and angle were automatically calculated using a self‐developed script/macro (developed by Dr Norbert Vischer) (Notes S1). Root growth velocity was calculated separately for day and night by dividing the particular root length over time.

### Root penetration assay

Agar plates were generated containing ½MS media with either 0.7% or 1.6% agar and three P_i_ concentrations (300, 625 or 1250 μM) using square petri dishes (15 × 15 cm). After solidifying, the top 2 cm of the agar medium was scooped out with a sterile scalpel and spatula, and replaced by pouring in ½MS medium with different P_i_ concentrations with 0.7% agar. After solidifying, the top 1 cm medium was again removed to create space for the shoot. Plates were put straight up and seeds pressed (1 mm) into the middle of the agar slab, after which the plates were placed vertically (90°) in the growth chamber using the above described conditions. After 6 d of growth, plates were photographed and the percentage of bending *vs* nonbending roots was determined.

### Statistical analysis

Statistical analyses were performed using GraphPad Prism 9. The Shapiro–Wilk test was applied for analysing the normal distribution of values. One‐way or two‐way analysis of variance (ANOVA) followed by Tukey's or Šídák's multiple comparison tests was used. If values were not normally distributed, the Kruskal–Wallis test followed by Dunn's multiple comparison tests were used. Different letters indicate significant differences between different treatments (*P* < 0.05). Asterisks indicate a statistically significant difference among different treatments. Significantly different means (*, *P* < 0.05; **, *P* < 0.01; ***, *P* < 0.001; ns, not significant) were separated by Tukey's NEJM.

## Results

### Arabidopsis roots do not exhibit phosphotropism

**Fig. 1:**
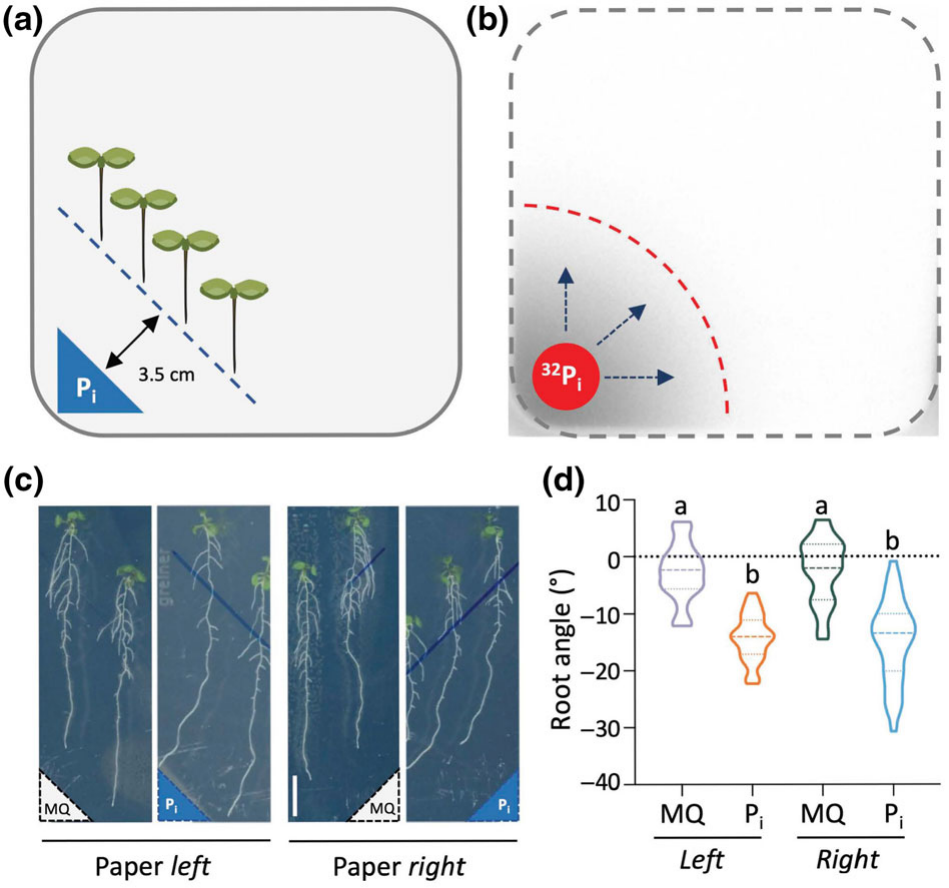
Arabidopsis root growth deviates to the left in response to increased P_i_ levels, independent of where the concentration gradient comes from. (a) Schematic diagram of P_i_‐tropism assay, with low‐P_i_ concentration in the agar medium and high P_i_ on a piece of filter paper (blue), placed at the left‐hand corner, 3.5 cm away from the seedlings. (b) Visualisation of P_i_ diffusion using radioactive ^32^P_i_. The ^32^P_i_ was spotted onto the filter paper and the radioactivity imaged 97 h later. (c) Phenotype of Col‐0 seedlings grown on low P_i_‐agar medium with filter paper containing high P_i_ or MQ water, either on the left‐ or right side of the agar plate, 6 d after transfer. (d) Quantification of root angles. The middle line represents the median, and the dotted line represents quartiles. Different letters indicate significant differences (*P* < 0.05) among treatments (One‐way ANOVA followed by Tukey HSD *post hoc* analysis, *F*_3,133_ = 48.48, *P* < 0.001). *n* = 25–49. Bar, 1 cm.

To investigate whether roots of *Arabidopsis thaliana* were phosphotropic, that is able to sense P_i_ and grow towards it, we transferred 4‐d‐old Arabidopsis (Col‐0) seedlings that grown on normal ½MS‐agar medium to ½MS medium containing low P_i_ (15 μM), and placed a small piece of filter paper in the bottom corner that contained either 40 μl of 100 mM phosphate buffer (K_2_HPO_4_/KH_2_PO_4_ mixture, pH 5.8) to create a P_i_‐concentration gradient, or MilliQ (MQ) water as control (Fig. 1a). Seedlings were positioned diagonally, *c*. 3.5 cm away from the filter paper, and subsequent growth was followed by scanning the agar plates daily. The diffusion of P_i_ from the dry filter paper into the agar was confirmed using radioactive ^32^P_i_ and Phosphoimaging (Fig. 1b), revealing a diffusion rate of *c*. 1 cm per day.

Application of P_i_ in the left‐hand lower corner changed the root growth direction towards the P_i_ source, which did not occur when MilliQ water without P_i_ was used (Fig. 1c). However, when the P_i_ source was placed in the opposite, right‐hand corner, roots were still found to redirect their growth to the left (Fig. 1c,d). Similar results were obtained when part of the low P_i_‐agar medium was replaced with a high P_i_‐agar medium (Fig. S3). While these results are incompatible with a phosphotropism response, they do show that roots sense P_i_ and change their direction in response to an increase in P_i_.

### Increasing KH_2_PO_4_ concentrations promote root skewing

To obtain a clearer picture of the dose‐dependency of the response, Arabidopsis Col‐0 seedlings were grown on ½MS medium containing a range of KH_2_PO_4_ concentrations. Standard ½MS medium contains 625 μM P_i_ but by using commercial MS medium without (W/O) P_i_ as basis, KH_2_PO_4_ concentrations could be varied. In these experiments, seedlings were grown for 9 d in a vertical, slightly tilted (70°) position (Fig. S1A).

**Fig. 2:**
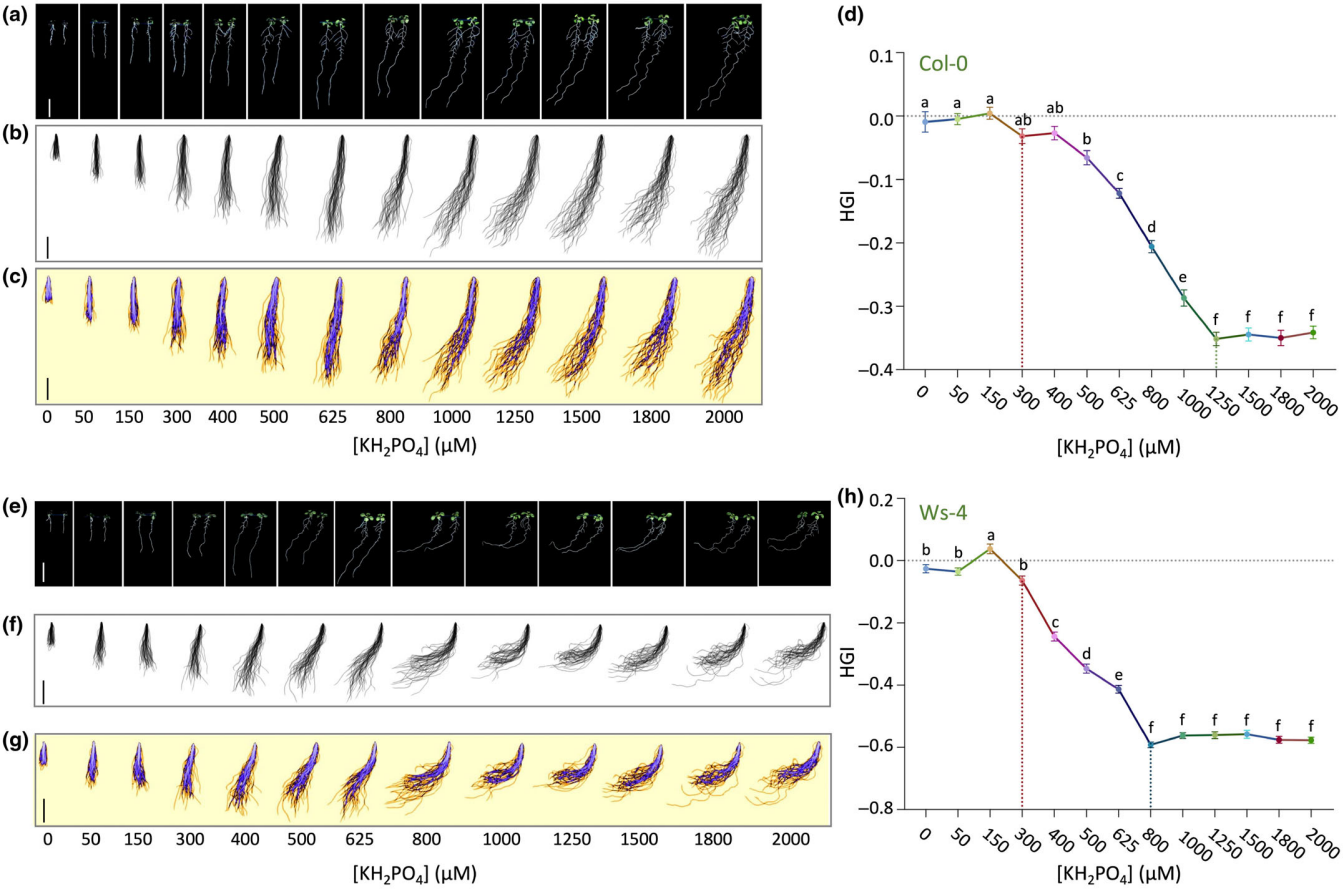
Increasing KH_2_PO_4_ concentrations promote root skewing in Arabidopsis ecotypes Col‐0 (a–d) and Ws‐4 (e–h). (a, e) Seedling phenotype after 9 d of growth on ½MS medium containing indicated KH_2_PO_4_ concentrations. (b, f) Projections of primary roots of 34–40 seedlings grown at indicated P_i_ concentration. (c, g) Same as panels b and f, but with the overlap indicated in purple. (d, h) Quantification of root skewing using horizontal growth index (HGI). Data represent means ± SE (*n* = 34–40). Different letters indicate significant differences (*P* < 0.05) among treatments using One‐way ANOVA followed by Tukey HSD *post hoc* analysis: *F*_12,479_ = 189.5, *P* < 0.0001 (d) or *F*_12,484_ = 415, *P* < 0.0001 (h). Bars, 1 cm.

As shown in Fig. 2(a–d), leftward skewing of roots increased with increasing KH_2_PO_4_ concentrations (Fig. 2a). To display multiple primary roots at the same P_i_ concentration, projections of 40–43 seedlings were made (Fig. 2b) and the overlap visualised in purple (Fig. 2c). The degree of skewing was measured by determining the horizontal growth index (HGI) (Fig. S1B); a flexible and sensitive quantitative descriptor of root angle phenotypes that is independent of morphometric parameters (Grabov *et al*., 2005; Vaughn & Masson, 2011). Negative HGI values indicate roots growing to the left; positive values mean roots would grow to the right. HGI values of Col‐0 roots started decreasing at 300 μM P_i_ and reached a minimum at 1250 μM P_i_ (Fig. 2d), which is the actual P_i_ concentration in the original recipe of Murashige & Skoog (MS) (Murashige & Skoog, 1962).

Arabidopsis ecotype, Wassilewskija (Ws) is notorious for its root skewing, though nobody has been able to explain why (Rutherford & Masson, 1996; Sedbrook & Kaloriti, 2008; Arieti & Staiger, 2020). To assess whether this enhanced‐skewing phenotype is related to P_i_, Ws‐4 seedlings were exposed to the same KH_2_PO_4_ concentration range (Fig. 2e–h). Ws‐4 seedlings showed a very strong, dose‐dependent root‐skewing phenotype, up to a point that roots almost grew horizontally (Fig. 2e–g). Similar to Col‐0, HGI values started decreasing at *c*.300 μM P_i_; however, Ws‐4 already reached its minimum at 800 μM P_i_, and with a much lower HGI (−0.58 compared to Col‐0 at −0.35; Fig. 2d,h). These results not only confirm that KH_2_PO_4_ promotes root skewing; they also demonstrate that there is genetic variation for this response.

### Phosphate is the main factor causing root skewing

**Fig. 3:**
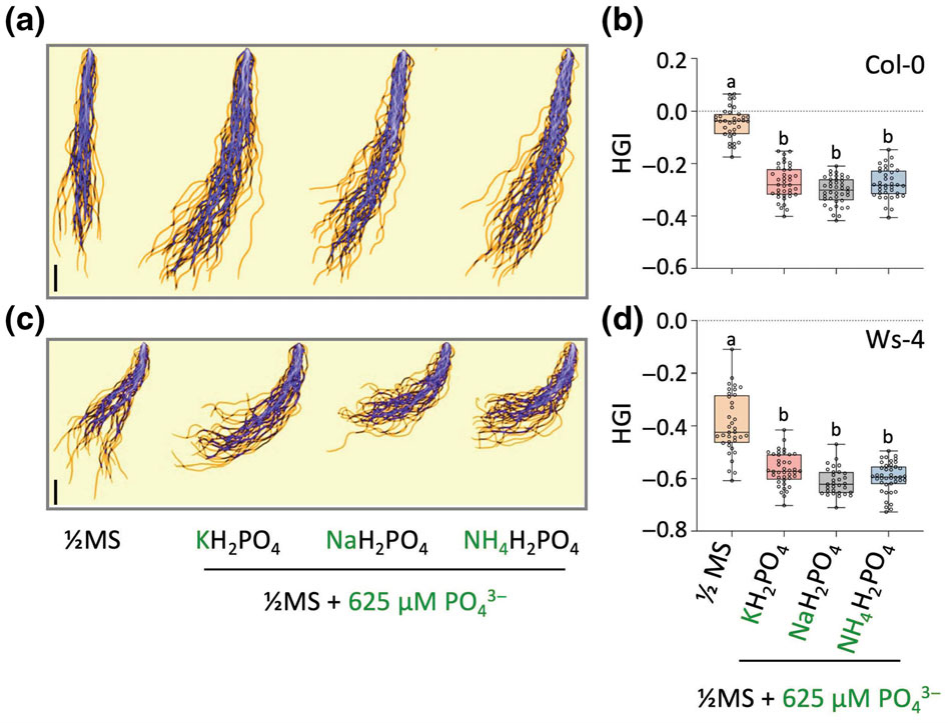
Phosphate is responsible for the induced root skewing in Arabidopsis. (a, c) Primary root projections and overlap (purple) of 9‐d‐old seedlings of Ws‐4 (a) and Col‐0 (c), grown on normal ½‐strength Murashige & Skoog medium (½MS) (containing standard 625 μM P_i_) or ½MS supplemented with 625 μM P_i_ of different cationic composition, that is K^+^, Na^+^, or NH_4_^+^. (b, d) HGI for Col‐0 (b) and Ws‐4 (d). The middle line represents the median and box limits indicate the 25^th^ and 75^th^ percentiles; whiskers span down to the minimum and up to the maximum values, and individual data points are represented by dots. One‐way ANOVA was performed to identify significant differences of Ws‐4 (*F*_3,144_ = 64.39, *P* < 0.0001) and Col‐0 (*F*_3,147_ = 155.3, *P* < 0.001) between treatments. Different letters indicate a statistically significant difference (*P* < 0.05) by Tukey HSD *post hoc* analysis (*n* = 31–40). Bars, 1 cm.

To assess whether it is indeed P_i_ that is responsible for the skewing response, we investigated the effect of other salts. Hereto, ½MS medium containing the standard 625 μM KH_2_PO_4_ was supplemented with 625 μM KH_2_PO_4_, NaH_2_PO_4_, or NH_4_H_2_PO_4_, or without extra P_i_ (control). As shown in Fig. 3, all three P_i_ salts clearly promoted root skewing compared to the control, for both Col‐0 (Fig. 3a,b) and Ws‐4 (Fig. 3c,d). By contrast, sulphate (SO_4_^2−^) or nitrate (NO_3_^−^) salts were not able to trigger skewing (Fig. S4), indicating the response was not simply anionic but depending on P_i_.

Recent studies have shown that the typical inhibition of primary root growth by P_i_ starvation appears to be dependent on the presence of Fe in the medium (Müller *et al*., 2015; Godon *et al*., 2019; Clúa *et al*., 2024; Maniero *et al*., 2024). Since P_i_ can interact with Fe, increased P_i_ concentrations could lower Fe availability, and hence, be an alternative cause for the observed skewing response. To investigate this, we tested whether increased Fe concentrations were able to reduce the PDS response when P_i_ was raised from 625 μM to 1250 μM. However, neither Fe^2+^ nor Fe^3+^, were able to reduce PDS (Fig. S5). Similarly, light has been shown to affect primary root‐growth inhibition under P_i_ starvation via photoreceptors CRY1 and CRY2 (Liu *et al*., 2017; Sakuraba *et al*., 2018; Gao *et al*., 2021).

To assess the effect of light on root skewing, a setup was constructed where only shoots were exposed to light and roots were kept in the dark (as much as possible; Fig. S6A,B). Though dark‐grown roots showed a slightly reduced skewing response compared to complete light, it was still clearly enhanced by P_i_, and the reduction was merely due to the reduced growth (Fig. S6C). Together these finding indicates that the P_i_‐triggered root skewing response is not due to ion charge, light or Fe and hence was termed, ‘phosphate‐dependent skewing’ (PDS).

To verify that the PDS response requires P_i_ uptake and is influenced by P_i_ signalling, we tested the P_i_ transport and ‐signalling mutants *pho1*, *pho2*, *phf1*, and *phr1phl1* under three different P_i_ conditions (Fig. S7A) (González *et al*., 2005; Aung *et al*., 2006; Bustos *et al*., 2010; Liu *et al*., 2012). Except for *pho1‐2* mutant, all other three mutants (*pho2*, *phf1* and *phr1phl1*) showed reduced PDS responses (Fig. S7B). These findings confirm that the PDS response requires P_i_ uptake and ‐signalling. Additionally, it suggests that root P_i_ concentration plays a more critical role than shoot P_i_ concentration and system P_i_ signalling of PDS response.

Since seedlings were grown on vertical, slightly tilted, agar plates, two additional parameters were considered to potentially affect PDS: (1) the concentration of the agar; and (2) the angle at which the tilted plates were standing. Agar is a natural product containing carbohydrate polymers and ions, and its water content will vary with its concentration, and this may affect the friction between root and agar gel, and hence, skewing response. Similarly, since roots are gravitropic, one can imagine that there are friction differences between plates standing at an angle of 45° or vertical (90°). Hence, three agar concentrations (0.75, 1.0, and 1.5%, w/w) were tested at our routine 70° angle, and three plate angles (45°, 70°, and 90°), using our routine 1% agar concentration. To validate the P_i_ dependency, three P_i_ concentrations were used, that is 300, 625, and 1250 μM KH_2_PO_4_. Lower plate angles indeed resulted in enhanced‐skewing responses (lower HGI values), and this response was again stronger for Ws‐4 than for Col‐0. Independent of the angle, however, skewing was strongly enhanced by P_i_ in all cases (Fig. S8A–H). Agar concentrations of 0.75% and 1% gave similar results, while 1.5% inhibited PDS, especially for Col‐0 (Fig. S9A–H).

### PDS dynamics

To investigate the skewing dynamics in more detail and over a longer time period, we used a custom‐built time‐lapse setup (Fig. S1D), allowing us to continuously image root growth for over a week. Images were taken every 10 min and were integrated into a video using ImageJ. Col‐0 (left) and Ws‐4 (right) seedlings were simultaneously analysed on the same plate at either 300 μM P_i_ or 1250 μM P_i_ (Videos S1 and S2, respectively).

**Fig. 4:**
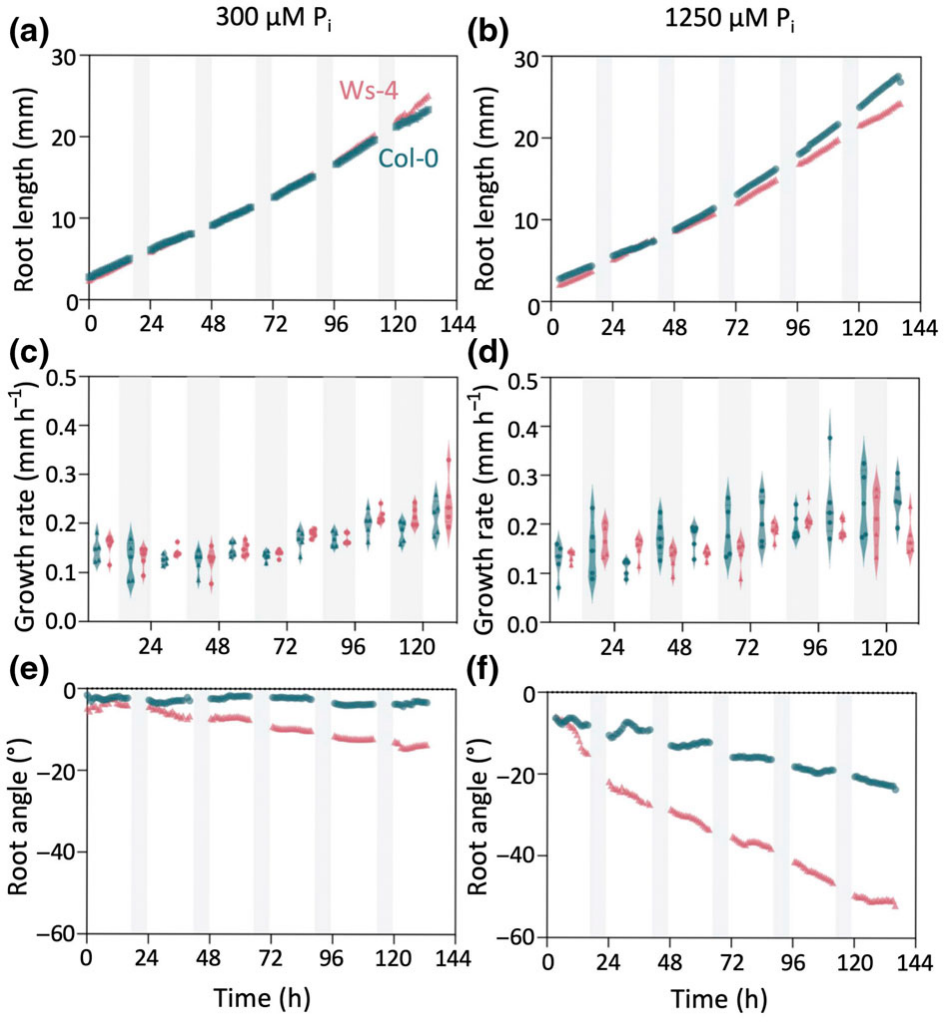
Time‐lapse of root growth and skewing responses in Col‐0 and Ws‐4 seedlings at 300 or 1250 μM P_i_. The growth of Arabidopsis Col‐0 and Ws‐4 seedlings were followed over a period of 6 d in a 16 h : 8 h, L : D regime. Images were captured every 10 min. Left panels (a, c, e) 300 μM P_i_, right panels (b, d, f) 1250 μM P_i_. (a, b) Primary root length, (c, d) growth rate, and (e, f) primary root angles. Values represent the means of five biological replicates of five seedlings. Blue symbols, Col‐0; red symbols, Ws‐4. See also Supporting Information Videos S1 and S2.

For every image, root length, growth rate, and root angle were analysed at 300 μM P_i_ (Fig. 4a,c,e) and 1250 μM P_i_ (Fig. 4b,d,f), excluding the dark periods (16 h : 8 h, light : dark regime). Quantification showed that the root length at 300 μM P_i_ increased similarly for both ecotypes, but that Col‐0 grew slightly longer roots at 1250 μM P_i_ (Fig. 4a,b). For both ecotypes, the growth rate was found to gradually increase at both P_i_ concentrations (Fig. 4c,d). The root angle patterns were initially dominated by root waving in both directions but after *c*. 25 h, a strong directional growth (skewing) became apparent, stronger for Ws‐4 and more dominant at the higher P_i_ concentration (Fig. 4e,f). Although images could not be captured during the 8 h dark period, interpolation of the data shows that Ws‐4 skewed during both day and night, while Col‐0 preferably skewed during the dark period, like growth itself (Fig. 4f).

### P_i_ also promotes lateral root skewing

**Fig. 5:**
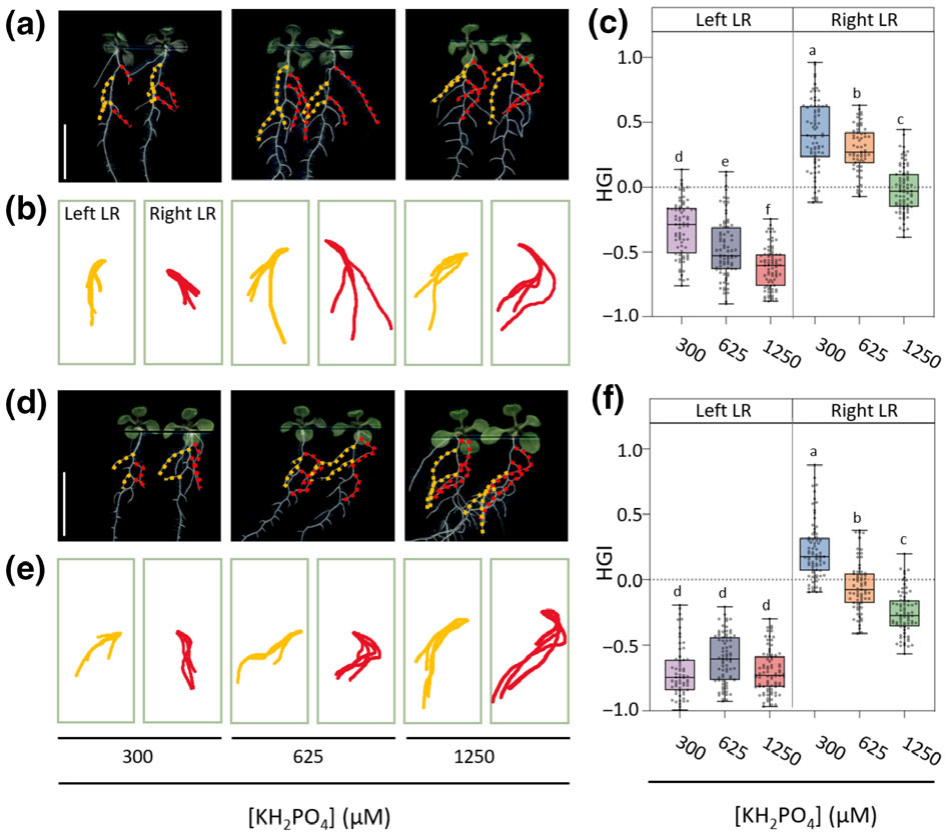
Increasing P_i_ concentrations promote skewing of lateral roots. Root system architecture of *Arabidopsis thaliana* seedlings, ecotypes Col‐0 (a) and Ws‐4 (d) grown at 300‐, 625‐, or 1250 μM P_i_ for 10 d. The first two lateral roots on the left (indicated in red) and on the right (indicated in yellow) were analysed for growth directions. (b, e) Projections of multiple left (red) or right (yellow) lateral roots of Col‐0 (b) or Ws‐4 (e). (c, f) Box plot analyses of HGI values for left‐ and right lateral roots of Col‐0 (c) or Ws‐4 (f). The middle line represents the median and box limits indicate the 25^th^ and 75^th^ percentiles; whiskers span down to the minimum and up to the maximum values, and individual data points are represented by dots. The one‐way ANOVA and Kruskal–Wallis test were performed to identify significant differences of Col‐0 (*F*_5,445_ = 297, *P* < 0.0001) and Ws‐4, respectively. Different letters indicate significant differences (*P* < 0.05) among treatments. Values represent lateral roots of 30–41 seedlings. Bars, 1 cm.

So far, attention was focused on the skewing behaviour of primary roots. Next, the susceptibility of lateral roots to P_i_ was investigated (Figs 5, S1C). For this, the RSA of 10‐d‐old seedlings was analysed using the same three P_i_ concentrations. The first two lateral roots on the left‐ (indicated in yellow) and right side (indicated in red), for both Col‐0 (Fig. 5a–c) and Ws‐4 (Fig. 5e,f), were analysed, and subsequent HGI values determined (Fig. 5c,f). Clearly, the lateral roots displayed a dose‐dependent PDS response too, and this was most evident for the lateral roots on the right side, as they even crossed the main root at the highest P_i_ concentration in both ecotypes, which already occurred for Ws‐4 at 625 μM. This dose‐dependency was also visible for the left lateral roots of Col‐0 (Fig. 5c), but not for Ws‐4, probably because the maximum horizontal growth of the left lateral roots was already reached at 300 μM P_i_ (Fig. 5f). Nonetheless, the results clearly show that not only primary roots but also lateral roots skew in a P_i_‐dependent manner.

### P_i_ increases epidermal cell file rotation

**Fig. 6:**
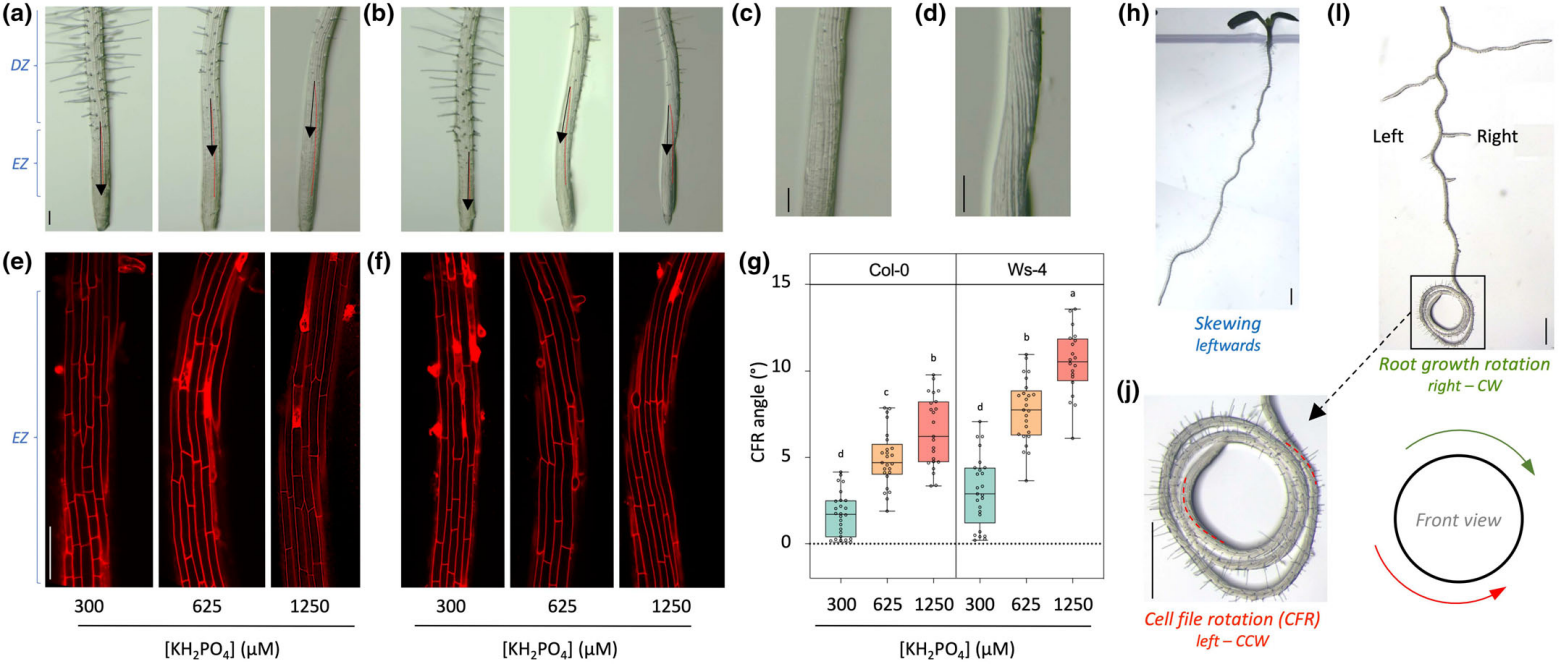
P_i_ promotes right‐handed root growth and left‐handed epidermal cell file rotation (CFR). (a, b) Stereomicroscopic images of root tips of Arabidopsis Col‐0 (a) and Ws‐4 (b) at 300‐, 625‐, or 1250 μM of P_i_. (c, d) Zoom‐in of elongation zone of Col‐0 (c) and Ws‐4 (d) root grown at 1250 μM P_i_. Black arrows indicate root growth direction; red dotted lines indicate CFR direction. (e, f) Confocal images of root elongation zone of propidium iodide‐stained Col‐0 (e) or Ws‐4 (f) seedlings, grown at indicated P_i_ concentrations. (g) Quantitative analysis of epidermal CFR angle at elongation zone of Col‐0 or Ws‐4 seedlings. Different letters indicate significant differences (*P* < 0.05) between treatments (One‐way ANOVA followed by Tukey HSD *post hoc* analysis, *F*_5,139_ = 73.92, *P* < 0.0001). Values represent three biological replicates with 6–11 seedlings analysed for each replicate. The middle line represents the median and box limits indicate the 25^th^ and 75^th^ percentiles; whiskers span down to the minimum and up to the maximum values, and individual data points are represented by dots. (h) Skewing direction of seedling (front view). (i, j) Stereomicroscopic image showing right‐handed (clockwise) root growth (i) and left‐handed (counterclockwise) epidermal cell file rotation (j) in the same image, captured from a Col‐0 seedling grown vertically for 4 d and was then placed horizontally for another 2 d, which causes roots to coil into their growth direction. Red dotted lines indicate the direction of the epidermal cell file (i). All images are front view. Bars: (a–f) 100 μm; (h–j) 1 mm.

Cell file rotation (CFR) is manifested as a twisting of the epidermal cell file along the root, visible at the base of the elongation zone (González *et al*., 2005; Mochizuki *et al*., 2005; Aung *et al*., 2006; Bustos *et al*., 2010; Liu *et al*., 2012). It is thought to be indicative of the circumnutation of the root, which has been suggested to cause skewing on hard‐agar surfaces (Rutherford & Masson, 1996; Yuen *et al*., 2005; Vaughn & Masson, 2011). To assess whether P_i_ affects CFR, roots of 7‐d‐old seedlings of both Col‐0 and Ws‐4, grown at 300, 625, or 1250 μM P_i_, were analysed using a stereomicroscope. For both ecotypes, a P_i_‐dependent effect on the epidermal CFR was observed, showing a stronger left‐handed/counterclockwise (CCW) rotation with higher P_i_ concentrations (Fig. 6a–d), which was again more evident for Ws‐4 (Fig. 6b,d) (compare black arrows indicating root growth direction with red dotted line indicating CFR direction). Staining the cell walls with propidium iodide (PI, red fluorescence) showed this even better, and confirmed the CCW CFR for both Col‐0 (Fig. 6e) and Ws‐4 (Fig. 6f) at higher P_i_ concentrations. Measuring the angle between root growth and epidermal cell file revealed significantly higher CCW CFR angles at higher P_i_ concentrations, with Ws‐4 showing the strongest effect (Fig. 6g).

To emphasise the connection between CFR and root growth direction, both movements were analysed in one and the same image using seedlings that were grown horizontally (Fig. 6i,j). This again confirmed that leftward skewing (Fig. 6h) correlates with right‐handed, clockwise (CW) root growth, and left‐handed, CCW epidermal CFR (Fig. 6i,j).

### PDS requires reorganisation of the microtubule cytoskeleton

**Fig. 7:**
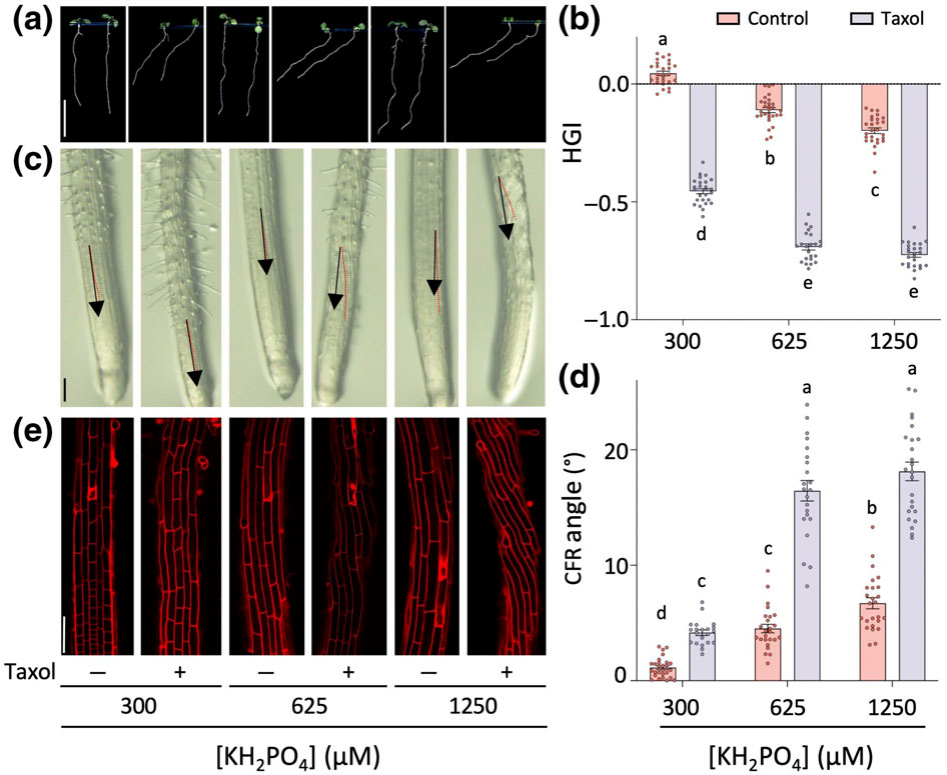
P_i_‐dependent root skewing is enhanced by microtubule‐stabilisation drug, Taxol. (a) Phenotype of Arabidopsis Col‐0 seedlings grown on indicated P_i_ concentrations with and without 1 μM Taxol after 7 d. Bar, 1 cm. (b) Quantification of HGI value of seedlings growing at different P_i_ concentrations with or without Taxol. Two‐way ANOVA was used to determine the significant differences between different P_i_ treatments (*F*_2,151_ = 300.8, *P* < 0.0001) and Taxol treatments (*F*_1,151_ = 3557, *P* < 0.0001). (c) Stereomicroscope images of the surface of roots exposed to different treatments. (d) Quantitative analysis of epidermal CFR angle at the base of the elongation zone at different P_i_ concentrations. Two‐way ANOVA was used to determine the significant differences between different P_i_ treatments (*F*_2,137_ = 179.8, *P* < 0.0001) and Taxol treatments (*F*_1,137_ = 393.7.1, *P* < 0.0001). (e) Confocal microscope images of propidium iodide‐stained elongation zone of root grown on different P_i_ concentrations. The data shown are means ± SE. Different letters indicate significant differences (*P* < 0.05) between treatments. All values represent two biological replicates with 11–15 seedlings analysed for each replicate. Bars, 100 μm.

Both root skewing and CFR have been linked to the organisation of the microtubule cytoskeleton, on the basis of mutant analyses (Thitamadee *et al*., 2002; Nakajima *et al*., 2004; Shoji *et al*., 2004; Molines *et al*., 2018; Buschmann & Borchers, 2020) and pharmacological drugs affecting microtubule dynamics (Oliva & Dunand, 2007; Hodge *et al*., 2009). To test whether PDS can be linked to a change in microtubule behaviour, Col‐0 seedlings were grown in the presence and absence of the microtubule‐stabilisation agent, taxol. Interestingly, taxol itself already induced a strong skewing response (Fig. 7a,b), as was observed earlier (Oliva & Dunand, 2007; Roy & Bassham, 2017; Yue *et al*., 2019). However, it is clear that the skewing response was still strongly enhanced by increasing P_i_ concentrations (Fig. 7a,b), and this was also reflected in the CFR (Fig. 7c–e). Similar results were obtained with propyzamide (Fig. S10), and both drugs revealed stronger effects on the PDS of Ws‐4 (Figs S11, S12).

**Fig. 8:**
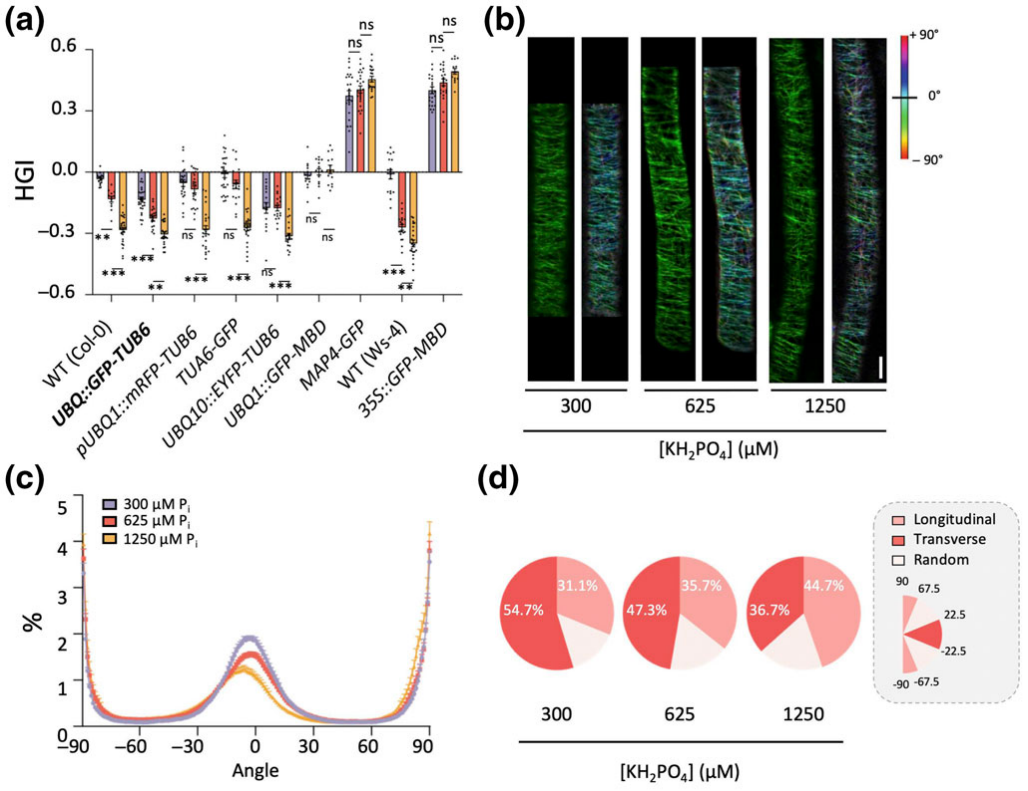
Effect of P_i_ on PDS and microtubule orientation in FP‐reporter lines. (a) Quantification of HGI value in different microtubule reporter lines, grown on different P_i_ concentrations. All values represent the means of two biological replicates with 6–15 seedlings analysed for each replicate. (b) Original confocal images (left, green) and ImageJ‐processed images (right, coloured) of 5‐d‐old *UBQ::GFP‐TUB6* seedlings. (c) Quantification of microtubule orientation by measuring multiple ROIs in cells grown at different P_i_ concentrations in Arabidopsis *UBQ::GFP‐TUB6*. (d) Distribution of microtubule orientation. Angles ranging from 22.5 to −22.5° were considered ‘Transverse’, while angles ranging from 67.5 to 90° and −67.5 to −90° were considered ‘Longitudinal’; all others were designated as ‘Random’. Values represent the mean of five biological replicates with 10–15 seedlings per condition. Data shown are means ± SE. Asterisks indicate a statistically significant difference (**, *P* < 0.01; ***, *P* < 0.001, ns, not significant) by two‐way ANOVA. Differences between 300 and 1250 μM were statistically significant (*P* < 0.001) for each line except for *UBQ1::GFP‐MBD*, which did not respond at all. Bar, 10 μm.

To further investigate the involvement of the microtubule cytoskeleton, various genetically encoded fluorescent‐reporter lines were used, including *UBQ::GFP‐TUB6* (Col‐0) (Le & Ambrose, 2018), *UBQ1::GFP‐MBD* (Col‐0) (Le & Ambrose, 2018), *TUA6‐GFP* (Col‐0) (Shaw *et al*., 2003; Vavrdová *et al*., 2020), *MAP4‐GFP* (Col‐0) (Marc *et al*., 1998; Komis *et al*., 2014; Tichá *et al*., 2020), *pUBQ1::mRFP‐TUB6* (Col‐0) (Ambrose *et al*., 2011; Novák *et al*., 2018), *UBQ10::EYFP‐TUB6* (Col‐0) (Sugiyama *et al*., 2017; Gunji *et al*., 2020) and *35S::GFP‐MBD* (Ws‐4) (Müller *et al*., 2007; Hamant *et al*., 2008; Hervieux *et al*., 2016). These markers consist of a fluorescent protein (FP) fused to either a microtubule‐binding protein (MAP4 or MBD) or an α‐ or β‐tubulin (TUA6 or TUB6), and are ectopically expressed through either 35S‐ or ubiquitin promoter. Strikingly, none of these marker lines behaved like wild‐type (WT) in terms of skewing, although most still showed the P_i_ sensitivity. Interestingly, reporters based on microtubule‐binding proteins (MAP4 or MBD) all skewed to the right, that is the opposite direction of PDS, and reacted less to P_i_, or not at all (i.e. *UBQ1::GFP‐MBD* (Le & Ambrose, 2018)) (Fig. 8a). All tubulin marker lines retained their skewing tendency to the left and were P_i_ sensitive (Fig. 8a), but for most (i.e. *pUBQ1::mRFP‐TUB6*, *TUA6‐GFP* and *UBQ10::EYFP‐MBD*), the FP signal in the root was too weak to visualise the orientation of the microtubules. Only line, *UBQ::GFP‐TUB6* (Le & Ambrose, 2018; Zhang *et al*., 2021) gave a reasonable fluorescent signal (Fig. 8b) and displayed a dose‐dependent PDS response (Fig. 8a). Hence, this line was used for the analysis of the orientation of the microtubules at distinct P_i_ concentrations, using the OrientationJ Analyses plugin tool of ImageJ, which gives false colours to different orientations (Fig. 8b). Classifying the microtubule orientation as ‘longitudinal’ (67.5 to 90° and −67.5 to −90°), ‘transverse’ (−22.5 to 22.5°) or ‘random’ (all other angles) showed that microtubules from seedlings grown at 300 μM P_i_ displayed a more *transverse* (horizontal) orientation, whereas those from 1250 μM P_i_ were more *longitudinal* (vertical) (Fig. 8d). Though these changes appear small, it is important to realise that the skewing response is a net result of hundreds of cells rotating over days, while the MT orientation imaging is from a single cell at a single time point.

### P_i_ affects directional root growth in a homogeneous environment

**Fig. 9:**
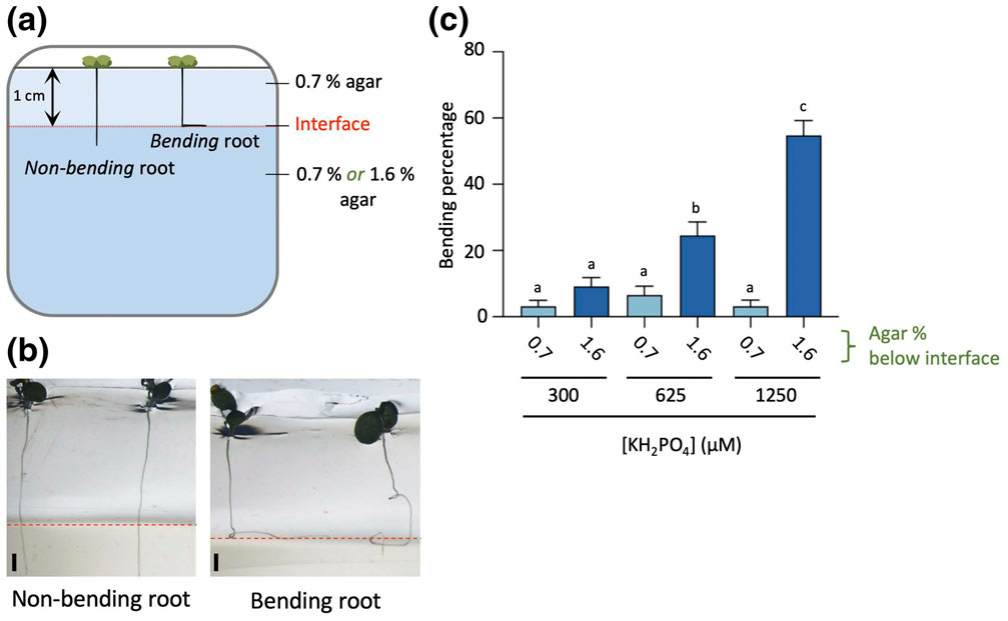
P_i_ affects root penetration power due to 3D skewing. To test whether P_i_ promotes PDS in a 3D‐agar environment, the bending of primary roots was analysed in a vertical two‐layered agar system. The first layer of 0.7% allows seed germination and root growth, while the second layer contains either 0.7% or 1.6% agar to score the penetration power of the roots. (a) Schematic drawing of the vertical two‐layered agar system. (b) Examples of Arabidopsis *bending* and *nonbending* roots. *Nonbending* roots directly penetrate the lower layer medium while *bending* roots bounch‐off the lower agar medium and grow along the interface. Left panel shows typical nonbending root at 300 μM P_i_ while right panels shows typical bending/skewing roots at 1250 μM (c) Quantification of roots showing a bending response at 300, 625 and 1250 μM P_i_. *X*‐axis indicates agar concentration of the lower layer (0.7% or 1.6%). Data shown are means ± SE. The Kruskal–Wallis test was performed to identify significant differences. Different letters indicate significant differences (*P* < 0.05) among treatments. All values represent two biological replicates. *n* = 91–126. Bars, 1 mm.

To investigate the potential effect of P_i_ on skewing in a homogeneous environment, a vertical (90°) two‐layered agar system was constructed (Fig. 9a). The top layer consisted of a soft (0.7%) agar, in which Col‐0 seeds were able to germinate and the roots to grow into, and a second layer of agar that either consisted of again the soft 0.7% agar (control), or a harder, 1.6% agar (Fig. 9a). We hypothesised that when roots would grow perfectly vertical, they would more easily penetrate the harder 1.6% agar layer than when roots would skew and hit the agar interface at an angle. In the latter case, penetration would be more difficult and roots were expected to bounce‐off the harder agar layer and grow along the surface, giving rise to ‘bended roots’ (Fig. 9b). As shown in Fig. 9(c), the number of roots bouncing‐off the harder agar layer, was significantly increased with increasing P_i_ concentrations, indicating that P_i_ affects the directional root growth when surrounded by agar.

## Discussion

### Phosphate‐dependent skewing (PDS)

**Fig. 10:**
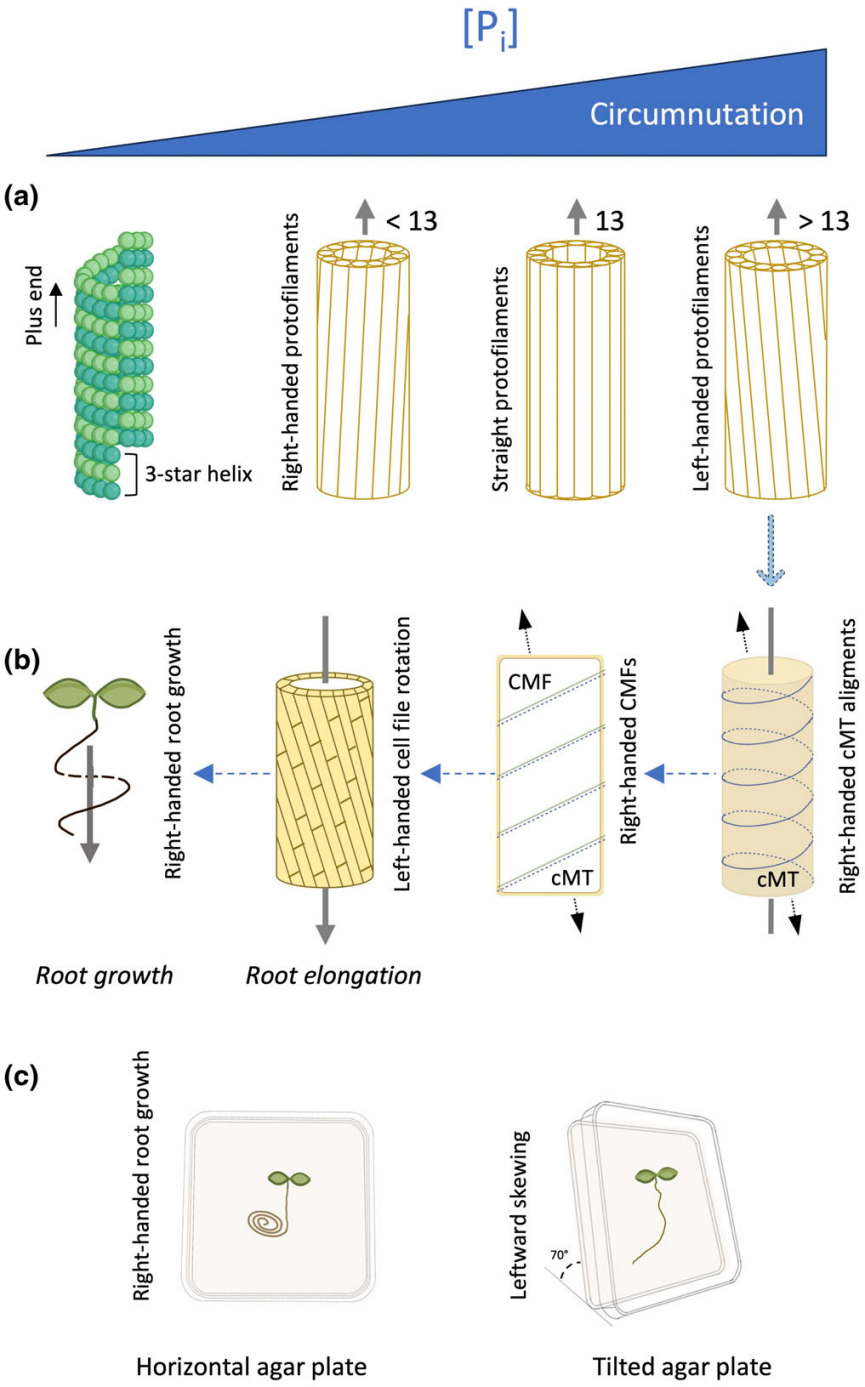
Model explaining right‐ and left‐handed directional movements of microtubules, cells and root during PDS in Arabidopsis. (a–c) Mechanism involves the rearrangement of microtubules affecting the CFR of epidermal cells in the elongation zone and rotational growth direction (circumnutation) of the root tip. Increased P_i_ concentrations promote a right‐handed shift of cortical microtubules (cMT) (a), which is followed by the right‐handed twisting of cellulose microfibrils (CMF) (b). This creates a biophysical torsion during cell elongation that triggers an increase in left‐handed CFR of epidermal cells in the root elongation zone (b), and promotes the subsequent rightward growth of the root (as seen on horizontal agar plates) and the leftward skewing on vertical plates (c). Figure is adapted from Zhou (2021).

In the present study, we demonstrate that *A. thaliana* roots (Col‐0) do not seem to exhibit phosphotropism, that is they do not grow towards P_i_ but rather show a clear P_i_‐specific directional growth response. On a horizontal agar plate, P_i_ triggers a CW (right‐handed) coiling of the root, which coincides with a CCW (left‐handed) epidermal CFR in the root elongation zone (Fig. 6). When the agar plate is tilted vertically in an angle of 70°, the CW rotation of the root manifests itself as a leftward‐skewing response, basically because the root is continuously pulling itself up to the left while making CW rotations (Figs 6, 10). The force that the root executes onto the agar surface to make these turns renders the response to be dependent on the angle of the tilted plate (gravitropism), though the P_i_ dependency of the whole process is clearly dominating (Figs S8, S9). Testing some P_i_ transport and ‐signalling mutants indicates that PDS involves both P_i_ uptake and signalling (Fig. S7). Hence, we termed the response, *Phosphate‐Dependent Skewing* (PDS). Our findings are supported by a recent study indicating that phosphate‐starved roots exhibit a significantly lower root skewing angle compared to phosphate‐replete roots (Matthus *et al*., 2020).

Both the primary and lateral roots exhibit PDS (Figs 5, S13), while no helical effects were found for hypocotyl, shoot or inflorescence (not shown), indicating that PDS is root‐specific. Differences in ecotypes Col‐0 and Ws‐4 indicate that there is a genetic base for PDS, as has been reported for skewing itself (Vaughn & Masson, 2011; Califar *et al*., 2020). Since PDS already starts at 300 μM, and most Arabidopsis researchers use ½MS medium that contains 625 μM P_i_, it is well possible that the majority, if not all, of the ecotypes with altered skewing responses, somehow differ in their P_i_ sensitivity. As such, future GWAS and/or QTL analyses will be interesting to identify potential molecular components of PDS.

### P_i_ tropism

There is a large body of literature showing that P_i_ availability strongly affects root growth and development (Gutiérrez‐Alanís *et al*., 2018; Bouain *et al*., 2019; Crombez *et al*., 2019). Plants respond to P starvation by altering the RSA to facilitate a more thorough exploration of soil P (de Bang *et al*., 2021). However, much is still unknown about the mechanisms that underlie P_i_ sensing and the changes it triggers in RSA. It has been suggested that roots display P_i_ tropism. Roots of *Lupinus albus* showed a positive chemotropic growth towards a di‐sodium phosphate solution, although authors did not determine whether the tropism was caused by sodium or phosphate (Newcombe & Rhodes, 1904), or whether this represented a PDS response (this paper). A recent study showed that an NH_4_^+^ gradient stimulated nutritropism in the lateral roots of rice (Yamazaki *et al*., 2020). P_i_ enhanced this nutritropism response by showing a coiled phenotype, which only occurred when NH_4_^+^ was present (Yamazaki & Fujiwara, 2022). Though rice roots did not show a phosphotropism response directly, it does show that P_i_ plays a role in the optimum growth direction, for example coiling response. Our work shows that Arabidopsis roots exhibit a PDS response rather than a phosphotropism response, and as such, the rice experiments may have to be revisited as they may reflect a ‘similar’ skewing phenotype. Additional investigation will be required to explore the effect of nonlimiting P_i_ levels on RSA in other plant species, to further deepen our understanding regarding the role of P_i_ in facilitating optimal plant growth through RSA modulation.

### P_i_ promotes root circumnutation

The helical movement of plant organs, called circumnutation, is a widespread phenomenon across the plant kingdom, and was already demonstrated in 1880 by Charles Darwin in his book, *The power of Movements in Plants* (Darwin & Darwin, 1880). Examples include the circumnutation of roots (*Quercus robur*), tendrils (pea), coleoptiles (rye, oat), shoots (bean), and hypocotyls (sunflower, that is heliotropism).

The typical root movements of Arabidopsis on tilted agar plates, that is skewing‐ (also called slanting), coiling‐ and waving, are believed to result from an interplay between circumnutation and gravitropism, with thigmotropism only having moderate effects on the final pattern that emerges (Migliaccio & Piconese, 2001; Oliva & Dunand, 2007; Roy & Bassham, 2014; Shih *et al*., 2014; Swarbreck *et al*., 2019; Sipos & Várkonyi, 2022; Porat *et al*., 2024). Roots can skew without gravity, however, as seen with gravitropic mutants as well as space flight experiments (Paul *et al*., 2012; Califar *et al*., 2020), while we found no significant effect of P_i_ on the gravitropic response of Arabidopsis roots and vice versa (Fig. S14). Such results imply a much stronger role for circumnutation in root skewing than for gravitropism. Recent simulation studies (Sipos & Várkonyi, 2022; Porat *et al*., 2024), confirm that root waving and coiling arise from a combination of active gravitropism and passive root–plane interactions, but that skewing requires an intrinsic twist/spiral from cell file rotation and circumnutation (Bastien & Meroz, 2016; Zhou, 2021; Sipos & Várkonyi, 2022; Porat *et al*., 2024). Our experimental data is in agreement with these findings, and suggests that P_i_ stimulates circumnutation, through an as yet unknown molecular mechanism.

### Role for microtubules

On the cellular level, microtubules are known to have a profound effect on helical growth movements (Buschmann & Borchers, 2020). Evidence for their participation in root skewing has been published earlier (Thitamadee *et al*., 2002; Nakajima *et al*., 2004; Shoji *et al*., 2004; Molines *et al*., 2018), and is confirmed here for PDS (Fig. 8). Arabidopsis mutants *lefty1* and *lefty2* exhibit reduced microtubule stability due to defects in α‐tubulins 4 and 6, and have an enhanced leftward‐skewing phenotype associated with right‐handed CFR and right‐handed cortical microtubule arrays (Thitamadee *et al*., 2002). By contrast, *spiral1‐* (*spr1*) and *spr2* mutants display left‐handed CFR, rightward‐skewing, and left‐handed cortical microtubule arrays (Nakajima *et al*., 2004; Shoji *et al*., 2004).

Microtubules are helical microfilaments consisting of α‐ and β‐tubulin dimers that are dynamically assembled and disassembled within cells through GTP‐binding and hydrolysis, which drives the direction and dynamic reorientation of the microtubule cytoskeleton (Wasteneys, 2002, 2004; Chen *et al*., 2016). On average, a microtubule helix is composed of 13 protofilaments, assembled around a hollow core, and this results in a relatively straight microtubule array (Fig. 10a). Modelling studies have indicated that when the number of protofilaments within the helix increases (> 13), the protofilaments start shifting to the left, which causes the cortical microtubules (cMTs) to rotate to the right (CW). Vice versa, when the number of protofilaments becomes < 13, a helical shift to the right is induced, which causes cMTs to rotate CCW (Fig. 10a) (Sui & Downing, 2010; Nakamura & Hashimoto, 2020). Hence, components that regulate or interfere with the microtubule dynamics, for example pharmacological inhibitors or overexpression of FP‐tagged tubulins or microtubule‐binding proteins, are likely to affect the number of tubulins within a helix as well as the polymerisation/depolarisation rate, potentially affecting the microtubule orientation. We propose that P_i_ directly or indirectly affects the cortical microtubule orientation by stimulating its CW rotation. Since the cellulose synthase complex follows the arrangement of the microtubules, the orientation of the cellulose microfibrils aligns with the microtubules (Fig. 10b) (Buschmann & Borchers, 2020; Zhou, 2021). The CW helical rotation of the cellulose fibers generates a physical torsion in the epidermal cells, which drives the successive CCW CFR, CW helical‐root growth, and leftward skewing (Fig. 10b,c) (Baskin, 2001; Thitamadee *et al*., 2002; Wasteneys, 2004; Yuen *et al*., 2005; Paredez *et al*., 2006; Lucas & Shaw, 2008; Chan, 2012). The transverse‐to‐longitudinal reorientation of cortical microtubules (Fig. 8b) is likely part of the PDS response, reflecting the enhanced elongation of the epidermal cells in the elongation zone.

Elucidation of the mechanisms underlying the effect of P_i_ on microtubule dynamics in the root elongation zone, affecting CFR and skewing, will require further research. This includes measuring important microtubule parameters, such as treadmilling speed, rates of polymerisation, depolymerisation at microtubule ends, bundling and severing, but also identifying proteins that directly or indirectly regulate the organisation of the microtubule cytoskeleton and of which the expression is affected by P_i_. Alternatively, PDS could be regulated by the concentration of ATP and/or GTP, affecting protein kinases and GTPases, which are known to regulate microtubule dynamics (Wasteneys, 2004).

### Additional potential mechanism underlying PDS

There are also mutants with altered skewing phenotypes that have no obvious relationship with the microtubules. For example, karrikin and auxin have been implicated in root skewing (Swarbreck *et al*., 2019; Villaécija‐Aguilar *et al*., 2019; Hu *et al*., 2021), while strigolactones are typically induced by low‐P_i_ conditions (Al‐Babili & Bouwmeester, 2015). We tested a number of mutants in the biosynthesis and/or signalling of these hormones but found no evidence for their participation in PDS (Fig. S15).

Alternatively, the P_i_ responsiveness of genes earlier associated with root skewing was checked (Table S1). Among 23 genes, only two, *RHM1 (RHAMNOSE BIOSYNTHESIS 1)* and *MIK2 (MALE DISCOVERER 1‐INTERACTING RECEPTOR LIKE KINASE 2)* exhibited significant changes, though differences were very small (Fig. S16). *RHM1* encodes a UDP‐L‐Rhamnose synthase involved in rhamnose biosynthesis, a major component of the pectin. KO mutants exhibit increased left‐handed cell file rotation and leftward root skewing on tilted agar plates, and this appears to be cell wall‐related rather than microtubules (Saffer *et al*., 2017). KO mutants of *MIK2* exhibit enhanced leftward skewing too (Van der Does *et al*., 2017). The observed downregulation would fit enhanced skewing but whether this helps in the PDS response remains to be established.

Since low P_i_ induces the formation of root hairs, and higher P_i_ concentrations suppress this, we considered whether root hairs would act as a physical barrier that slows down circumnutation, and hence would reduce PDS under low P_i_. However, no effect on skewing and CFR was reported for various root hair defective mutants (*rhd2*, *rhd4*, and *rhd6*), while these responses were completely suppressed in *rhd3* (Yuen *et al*., 2005). Whereas these results strongly indicate that the effects of P_i_ on root hair formation and PDS are unrelated, they do point to an important role for RHD3 in skewing and CFR. Recent data showed that RHD3 is a dynamin‐like atlastin GTPase, involved in the membrane fusion of ER tubules and vesicular trafficking between ER and Golgi (Sun *et al*., 2020a,b). Interestingly, Armadillo‐repeat kinesin1 (ARK1) interacts with RHD3 to move ER via the plus‐end of microtubules (Sun *et al*., 2020b). Further research should show whether GTP, vesicular transport, and microtubules are indeed involved in PDS.

In a QTL study on skewing, no genes were positively identified, but the authors did point out an interesting region containing two candidate genes encoding *cis*‐prenyltransferases, which are part of the dolichol−/dolichyl phosphate biosynthesis pathway used for GPI‐anchoring and glycosylation of proteins important in cell wall assembly and composition (Vaughn & Masson, 2011). *Skewed 5* (*SKU5*) encodes a GPI‐anchored protein and *sku5* mutants exhibit a strong skewing phenotype (Sedbrook *et al*., 2002). Recently, SKU5 was identified as a multi‐copper oxidase, coordinating root cell wall formation through apoplastic redox reactions (Chen *et al*., 2023). The *SKU5* gene is strongly expressed in expanding tissues and belongs to a 19‐member gene family, designated *SKS* (SKU5 Similar) (Chen *et al*., 2023), of which SKS11 and SKS12 have recently been shown to play a role in pollen tube integrity, −growth and ‐guidance (Duan *et al*., 2022). COBRA, another GPI‐anchored protein, affects patterning of cellulose microfibrils (Roudier *et al*., 2005) and mutant seedlings also exhibit increased root skewing (Roudier *et al*., 2005; Ambrose *et al*., 2011; Duan *et al*., 2022; Chen *et al*., 2023). All this points to a role for the cell wall in skewing. This makes sense, as cell wall composition and rigidity will affect the friction between the root tip and the agar medium and its ability to rotate. It remains as yet unclear whether P_i_ can affect this.

### Physiological consequences

After cell division in the meristematic root zone, most of the root growth occurs through cell expansion in the elongation zone where the cell wall is still relatively flexible. Changes in the helical orientation of the microtubule arrays during cell expansion will have a strong effect on CFR (Nakajima *et al*., 2004; Mochizuki *et al*., 2005), which is then fixed in the differentiation zone through rigidification of the cell wall by reactive oxygen species (lignification) and pectin demethylation (Yuen *et al*., 2005; Vaahtera *et al*., 2019; Rui & Dinneny, 2020; Zhang & Zhang, 2020). Cells in the root elongation zone were typically longer when grown at higher P_i_ concentrations (Fig. 8b), which may potentially contribute to the skewing response (more rotation, more torsion over a longer distance). However, since Col‐0 and Ws‐4 roots exhibited similar growth rates at 300 μM and 1250 μM P_i_, while skewing was greatly different, we conclude that it is really the stimulating effect of P_i_ on circumnutation rate and/or amplitude that is driving PDS, rather than an effect of P_i_ on root growth.

We tried to investigate the effect of P_i_ on root growth in soil but the root system of Arabidopsis was too fragile to visualise any effect on the 3D‐RSA. In a homogeneous agar environment, roots did show a dose‐dependent effect of P_i_ on their ability to penetrate a harder agar layer or to bounch‐off because of the increased root angle by P_i_ (Fig. 9). However, there may be other explanations for the same experiment (e.g. sensitivity to mechanical hindrance, mechanosensing) so for sure this should be investigated in much more detail. Nonetheless, in soil, such a P_i_‐dependent mechanism would keep roots closer to the top soil where P_i_ concentrations are higher due to its strong negative charge. This also holds true for the lateral roots: with an increase in root rotation, they would also stay closer to the soil surface where the P_i_ resides. Such response is in stark contrast to the RSA changes induced by P_i_ starvation, where primary root growth is inhibited, and lateral root growth and root hair formation stimulated to increase the net surface area for P_i_ uptake (Péret *et al*., 2011; Crombez *et al*., 2019). While P_i_ starvation has been recognised as a crucial cue in root development, the discovery of PDS is an exciting addition, and may emphasise the general importance of P_i_ for plants. Benfey's lab recently showed the relevance of circumnutation for root penetration of rice (Taylor *et al*., 2021), but the role of P_i_ was not addressed. Testing the PDS response in soil will also be crucial. It will be challenging to visualise Arabidopsis roots in soil, but using luminescent roots (Rellán‐Álvarez *et al*., 2015) or X‐ray scanning (Teramoto *et al*., 2020) could possibly visualise the effect of different P_i_ conditions on RSA.

In future work, we will use the natural genetic variation in PDS in Arabidopsis to identify and characterise the key molecular components that underlie PDS and circumnutation. This will further deepen our understanding of how roots grow, penetrate soils, and respond to P_i_, also in other plant species. Such information could turn out to be important for breeding of crops with improved P‐use efficiency.

## Competing interests

None declared.

## Author contributions

HS, HJB and TM designed the experiments, HS performed all experiments and data analyses, HS and TM constructed the figures, and HS, HJB and TM wrote the manuscript.

## Supporting information


**Dataset S1** Source data in this study.

**Fig. S1** Schematic diagram of root skewing setup and measurement.
**Fig. S2** Visualisation of P_i_ gradient using radioactive ^32^P_i_.
**Fig. S3** Arabidopsis root grows to the left in response to increased P_i_ levels, independent of where the P_i_‐concentration gradient comes from.
**Fig. S4** Phosphate, but not sulphate or nitrate, induces root skewing.
**Fig. S5** PDS response is not caused by Fe^2+^ or Fe^3+^.
**Fig. S6** Effect of light on root skewing.
**Fig. S7** PDS response is triggered by external P_i_ concentration, by influencing both P_i_ uptake and P_i_ signalling pathway.
**Fig. S8** P_i_‐dependent root skewing is affected by plate angle.
**Fig. S9** P_i_‐dependent root skewing is affected by agar concentration.
**Fig. S10** Effect of taxol on P_i_‐dependent skewing of Ws‐4 roots.
**Fig. S11** Effect of microtubule‐destabilisation drug propyzamide on PDS in Col‐0 roots.
**Fig. S12** Effect of propyzamide on PDS in Ws‐4 roots.
**Fig. S13** Heatmap showing the different expression levels of skewing‐related genes in Arabidopsis Col‐0 roots at different P_i_ concentrations.
**Fig. S14** P_i_ promotes right‐handed growth of both primary‐ and lateral roots on horizontal agar plates.
**Fig. S15** Gravitropism response is not affected by P_i_ and PDS response is not depending on gravitropic angle.
**Fig. S16** Auxin and strigolactone appear not to be involved in PDS signalling.
**Methods S1** Material and Methods used to obtain supporting figures and data.

**Notes S1** A self‐developed script/macro used in the time‐lapse.
**Table S1** Mutants with skewing phenotype from earlier studies.

**Video S1** Time‐lapse of root growth and skewing of Col‐0 (left) and Ws‐4 (right) seedlings at 300 μM P_i_.

**Video S2** Time‐lapse of root growth and skewing in Col‐0 (left) and Ws‐4 (right) seedlings at 1250 μM P_i_.
Please note: Wiley is not responsible for the content or functionality of any Supporting Information supplied by the authors. Any queries (other than missing material) should be directed to the *New Phytologist* Central Office.

## Data Availability

All data generated and/or analysed during this study are included in this paper and its Supporting Information files (Figs S1–S16; Methods S1; Table S1; Videos S1, S2; Notes S1). Materials used in this study are available from the corresponding author on request. Source data (Dataset S1) are provided with this paper.
