# Supplementary material for: Phosphate promotes Arabidopsis root skewing and circumnutation through reorganisation of the microtubule cytoskeleton
Source: New Phytol. 2024 Oct 3;244(6):2311–25. doi: 10.1111/nph.20152 (PMC11579438; doi:10.1111/nph.20152)
Supplement: Supplementary file 2 — Fig. S1 Schematic diagram of root skewing setup and measurement. Fig. S2 Visualisation of Pi gradient using radioactive 32Pi. Fig. S3 Arabidopsis root grows to the left in response to increased Pi levels, independent of where the Pi‐concentration gradient comes from. Fig. S4 Phosphate, but not sulphate or nitrate, induces root skewing. Fig. S5 PDS response is not caused by Fe2+ or Fe3+. Fig. S6 Effect of light on root skewing. Fig. S7 PDS response is triggered by external Pi concentration, by influencing both Pi uptake and Pi signalling pathway. Fig. S8 Pi‐dependent root skewing is affected by plate angle. Fig. S9 Pi‐dependent root skewing is affected by agar concentration. Fig. S10 Effect of taxol on Pi‐dependent skewing of Ws‐4 roots. Fig. S11 Effect of microtubule‐destabilisation drug propyzamide on PDS in Col‐0 roots. Fig. S12 Effect of propyzamide on PDS in Ws‐4 roots. Fig. S13 Heatmap showing the different expression levels of skewing‐related genes in Arabidopsis Col‐0 roots at different Pi concentrations. Fig. S14 Pi promotes right‐handed growth of both primary‐ and lateral roots on horizontal agar plates. Fig. S15 Gravitropism response is not affected by Pi and PDS response is not depending on gravitropic angle. Fig. S16 Auxin and strigolactone appear not to be involved in PDS signalling. Methods S1 Material and Methods used to obtain supporting figures and data. [file NPH-244-2311-s005.docx]

**New Phytologist Supporting Information**

**Article title:** Phosphate promotes Arabidopsis root skewing and circumnutation through reorganisation of the microtubule cytoskeleton

**Authors:** Hui Sheng, Harro Bouwmeester, and Teun Munnik^*^

**Article acceptance date:** 5 September 2024

**PDF file includes:**

- Methods S1
- Table S1
- Figs S1-S16
- Legends Videos S1 & S2
- Legends Dataset S1
- References S1

**Methods S1**

*Plant materials*

To validate the effect of auxin and strigolactone signaling in PDS, *A. thaliana* Col-0 mutants were used. For auxin, these include *tir1, afb1, tir1afb1,* and *tir1afb1afb2*, all kindly provided by Remko Offringa (Leiden University). For the involvement of strigolactones, *max1-1, max3-9, max4-1, d27-1, max2-1, d14-1, cyp706* (SALK_053959C), *ubox* (SALK_014138) mutants were tested. Phosphate transport and signaling mutants, *pho1-2* and *pho2* were kindly provided by Tzyy-Jen Chiou (Agricultural Biotechnology Research Center, Taiwan); *phf1* and *phf1phr1* were kindly provided by Laurent Nussaume (Université Aix Marseille, France); *pho1-2* and *phr1phl1* were kindly provided by Nicolaus von Wirén (Leibniz-Institut (IPK), Germany) (Hamburger *et al.*, 2002; González *et al.*, 2005; Aung *et al.*, 2006; Bustos *et al.*, 2010; Liu *et al.*, 2012).

*P_i_ gradient through agar replacement*

Sterilized Col-0 seeds were sown on square petri dishes (12 ×12 cm) containing 40 ml ½MS medium with 1% (w/v) sucrose, 1% (w/v) Daishin agar (Duchefa), and set to pH 5.8 (KOH). Plates were stored at 4°C in the dark for two days and then vertically (70°) placed in a growth chamber at 22°C, with long-day photoperiod (16 h light / 8 h dark). After 5 days, seedlings were transferred to ½MS agar plates with either 10 μM (low P_i_) or 625 μM P_i_ (standard P_i_ concentration of ½MS), with their root tips exactly 2 cm away from a diagonal line that was drawn on the back from corner to corner, dividing the plate in half. After two days of incubation, the lower diagonal part of the agar medium was scoped out and replaced by regular ½MS medium, by pipetting in fresh liquid ½MS agar medium (~50°C). Plates were left to solidify and dry for 20 min, sealed with Leukopor tape, and transferred back to the growth chamber, vertically at 70° (see Suppl. Fig S3).

*Gravitropic response assay*

Sterilized seeds were sown on ½MS plates containing 300-, 625-, or 1250 μM P_i_ and grown for 4 days. Root tip locations were then recorded and the plates rotated for 90°, either clockwise or counterclockwise, or not rotated at all (control). Root tips were recorded every 24 h for 4 days. Angles between the vertical gravitropic axis and root axis were measured using ImageJ.

*Root growth response on horizontal agar plates*

To test the growth behavior on horizontal agar plates, sterilized seeds were sown on ½MS plates containing 300-, or 2500 μM P_i_ and grown for 4 days. Root tip locations were recorded and plates placed horizontal for 6 days, and scanned 1-, 2-, 3- and 6-days after the horizontal transfer.

*Root growth in dark setup*

To investigate the effect of light on root growth behaviour, sterilized seeds were sown on ½MS plates containing 300-, 625- or 1250 μM P_i_. The front side of the plates, from the sowing line down to the bottom, was covered with sterilized aluminum foil to prevent light exposure. Additionally, the back side of the plates were covered with black plastic film to ensure complete darkness. For the rest, seedlings were grown as described in the main manuscript.

*Gene expression analysis*

Sterilized Arabidopsis Col-0 seeds were sown on ½MS plates containing 300-, 625-, or 1250 μM P_i_. Root samples were collected after 9 days and total RNA extracted using the NucleoSpin RNA Plant Kit (MACHEREY-NAGEL). RNA-seq analysis was performed and heatmap was plotted by https://www.bioinformatics.com.

*Statistical analysis*

Statistical analyses were conducted using GraphPad Prism 10. The boxplot features a central line representing the median, with the box boundaries corresponding to the 25th and 75th percentiles. Whiskers extend to the minimum and maximum values, and individual data points are depicted as dots.

**Table S1**

**Supplementary Table S1: Mutants with skewing phenotype in previous studies.**

| **Gene ID** | **Gene symbol** | **Mutant** | **Description** | **References** |
| --- | --- | --- | --- | --- |
| AT1G24460 | *TNO1* | *tno1* | TGN-Localized SYP41 interacting protein | (Roy & Bassham, 2017) |
| AT1G01950 | *ARK2* | *ark2* | Armadillo repeat kinesin 2 | (Sakai *et al.*, 2008) |
| AT5G60920 | *COB* | *cob* | Glycosylphosphatidylinositol-anchored protein | (Yuen *et al.*, 2005; Roudier *et al.*, 2005) |
| AT3G13870 | *RHD3* | *rhd3* | Root hair defective 3 with GTP-binding motifs | (Yuen *et al.*, 2005) |
| AT5G28646 | *WVD2* | *wvd2-1* | Wave-dampened 2 | (Yuen *et al.*, 2003) |
| AT3G47690 | *EB1A* | *eb1a* | Microtubule end binding protein EB1A | (Bisgrove *et al.*, 2008) |
| AT5G62500 | *EB1B* | *eb1b* | Microtubule end binding protein EB1B | (Bisgrove *et al.*, 2008) |
| AT5G67270 | *EB1C* | *eb1c* | Microtubule end binding protein EB1C | (Bisgrove *et al.*, 2008) |
| AT3G51550 | *FERONIA* | *fer* | PM localized receptor-like kinase | (Shih *et al.*, 2014) |
| AT5G44610 | *MAP18/PCAP2* | *map18/*  *pcap2* | Microtubule-associated protein 18/Plasma membrane-associated Ca^2+^-binding protein-2 | (Wang *et al.*, 2007; Kato *et al.*, 2019) |
| AT4G27060 | *TORTIFOLIA1* | *tor1/spr2* | Plant-specific microtubule-associated protein | (Buschmann *et al.*, 2004) |
| AT3G04630 | *WDL1* | 35S:*WDL1* | WVD2-like protein with KLEEK domain | (Yuen *et al.*, 2003) |
| AT1G50890 | *SP2L* | *sp2l* | Microtubule associated protein | (Yao *et al.*, 2008) |
| AT4G08850 | *MIK2* | *mik2* | MDIS1-interacting receptor-like kinase 2 | (van der Does *et al.*, 2017) |
| AT1G78570 | *RHM1* | *rhm1* | Rhamnose biosynthesis 1 | (Saffer *et al.*, 2017) |
| AT2G03680 | *SPIRAL1* | *spr1/sku6* | Plant specific protein with repeated motif | (Furutani *et al.*, 2000) |
| AT4G12420 | *SKU5* | *sku5* | GPI-anchored, multicopper oxidase | (Sedbrook *et al.*, 2002) |
| AT5G37770 | *CML27* | *cml27-2/*  *tch2* | Calmodulin-like 24 | (Wang *et al.*, 2011) |
| AT3G04080 | *APY1* | *apy1* | Golgi-localized integral membrane enzyme | (Yang *et al.*, 2015) |
| AT5G18280 | *APY2* | *apy2* | enzyme with ATPase and ADPase activity | (Yang *et al.*, 2015) |
| AT3G17850 | *IREH1* | *ireh1* | Ncomplete root hair elongation 1 | (Yue *et al.*, 2019) |
| AT3G51770 | *ETO1* | *eto1* | Ethylene overproducer 1 | (Yuen *et al.*, 2005) |
| AT5G12990 | *CLE40* | *cle40* | Clavata3/ESR-related 40 | (Hobe *et al.*, 2003) |

**Figures S1-S16**

**Supplementary Figure S1: Schematic diagram of root skewing setup and measurement.**

(A) Side view of the setup for the root skewing assay on tilted growth medium. (B) Schematic representation of measuring the level of skewing through the horizontal growth index (HGI=Lx/L). Basically, it analyses how much the root tip deviates from the vertical axis (θ), via L, root length, and the measured length of the idealized root response (L_c_). The displacement of the root tip along the y-axis (L_y_) and the horizontal root-tip deviation (L_x_) are calculated in Excel. Image is adapted from Roy and Bassham (2017). (C) Schematic diagram of HGI analysis of left- and right lateral root (LR). (D) Drawing of the home-made imaging device for time-lapse analyses of Arabidopsis root growth on tilted agar plates in a growth chamber. Device holds an iPhone SE with attached macro lens (f = 60 mm) in front of the agar plate at an angle of 70°.

**Supplementary Figure S2: Visualization of P_i_-gradient using radioactive ^32^P_i_.**

Radioactive ^32^P_i_ was pipetted on a piece of filter paper that, after drying, was placed onto the agar medium in the left-hand bottom corner. The agar plate was subsequently scanned for radioactivity through Phosphoimaging after: (A) 1 h, (B) 22 h, (C) 50 h, (D) 73 h, (E) 97 h, and (F) 121 h.

**Supplementary Figure S3: Arabidopsis roots grow to the left in response to increased P_i_ levels, independent of where the P_i_-concentration gradient comes from.**

(A) Schematic diagram of P_i-_tropism assay, where half of the diagonal of low P_i_ (10 μM) agar medium was replaced normal ½MS agar medium (625 μM P_i_). (B) Root growth of Col-0 seedlings, 6 days after replacing the lower part of the agar medium. (C) Quantification of root angle. The middle line represents the median, the dotted line represents quartiles. An unpaired t-test (two-tailed) was used to identify significant differences between treatments. N=45-54. Scale bar = 1 cm.

**Supplementary Figure S4: Phosphate, but not sulfate or nitrate, induces root skewing.**

(A, C) Primary root projections and overlap (purple) of 9-day-old seedlings of Ws-4 (A) and Col-0 (C), grown on ½MS medium or ½MS medium supplemented with 625 μM KH_2_PO_4_, K_2_SO_4_, KHSO_4_ or NH_4_NO_3_. (B, D) HGI value for Ws-4 (B) and Col-0 (D). One-way ANOVA was performed to identify significant differences of Ws-4 (*F*_4,115_ = 13.13, *p* < 0.001) and Col-0 (*F*_4,193_ = 29.13, *p* < 0.001) between treatments. Different letters indicate a statistically significant difference (*p* < 0.05) by Tukey HSD post-hoc analysis (n = 19-43). Scale bar = 1 cm

**Supplementary Figure S5: PDS response is not caused by Fe^2+^ or Fe^3+^.**

Seedlings of Col-0 were grown for 9 days on control ½MS medium (containing standard 625 μM KH_2_PO_4_ and 50 μM FeNaEDTA) or ½MS medium containing 1250 μM KH_2_PO_4_ with or without extra reduced or oxidized Fe forms, i.e. either FeSO_4_·7H_2_O (50 μM) or FeCl_3_ (50 or 300 μM), after which HGI values were determined. One-way ANOVA was used to determine the significance between treatments. Different letters indicate a statistically significant difference (*p* < 0.05) by Tukey HSD post-hoc analysis (n = 36-78).

Question was whether the increase of P_i_ would decrease the available Fe, and whether the latter is the actual cause of PDS. We therefore doubled- or increased the amount of Fe by 6-fold in standard ½MS medium. Seedlings showed a clear PDS response when P_i_ was raised from 650 μM to 1250 μM while extra Fe could not reverse this.

**Supplementary Figure S6: Effect of light on root skewing.**

(A) Experimental setup of how roots of seedlings were grown in the dark (or shaded). (B) Zoom-in of 4-day-old Col-0 seedling roots grown in the dark. (C) HGI value of 9-day-old Col-0 roots under light and dark conditions. One-way ANOVA was used to determine the significance between different treatments. Different letters indicate a statistically significant difference (*p* < 0.05) by Tukey HSD post-hoc analysis among different treatments (n = 22-52). Clearly, both setups showed P_i_-dependent root skewing.

**Supplementary Figure S7: PDS response is triggered by external P_i_ concentration, by influencing both P_i_ uptake and P_i_ signalling pathway.**

Phosphate transport and signalling mutants (*pho1*, *pho2*, *phf1*, and *phr1phl1*) seedlings were grown for 9 days at three different P_i_ concentrations: 300-, 625-, or 1250 μM KH_2_PO_4_. (A) Table summarizing the functions of the mutants, P_i_ uptake rate, and cellular P_i_ content in root and shoot. (B) HGI value of the mutants at different P_i_ concentrations. One-way ANOVA was used to determine the significance between different treatments. Different letters indicate a statistically significant difference (*p* < 0.05) by Tukey HSD post-hoc analysis among different treatments.

**Supplementary Figure S8: P_i_-dependent root skewing is affected by plate angle.**

Seedlings were grown for 9 days at 300-, 625-, or 1250 μM P_i_ at a plate angle of 45°, 70° or 90° (A-D) Col-0; (E-H) Ws-4. (A, E) Seedling phenotype, (B, F) Projection and overlap (purple) of primary roots of 28-44 seedlings, (C, G) HGI values, (D, H) root length. Two-way ANOVA was used to determine the significance between different plates angle of Col-0 (HGI (C): *F*_2,297_ = 41.72, *p* < 0.001; root length (D): *F*_2,297_ = 37.17, *p* < 0.001) and Ws-4 (HGI (G): *F*_2,316_ = 32.66, *p* < 0.001; root length (H): *F*_2,316_ = 124.8, *p* < 0.001); the significant differences between different P_i_ concentrations of Col-0 (HGI (C): *F*_2,297_ = 641.1, *p* < 0.001; root length (D): *F*_2,297_ = 466.7, *p* < 0.001) and Ws-4 (HGI (G): *F*_2,316_ = 572.2, *p* < 0.001; root length (H): *F*_2,297_ = 42.54, *p* < 0.001). Different letters indicate a statistically significant difference (*p* < 0.05) by Tukey HSD post-hoc analysis among different treatments (n = 28-44). Scale bar = 1 cm.

**Supplementary Figure S9: P_i_-dependent root skewing is affected by agar concentration.**

Seedlings were grown for 9 days at 300-, 625-, or 1250 μM P_i_ with either 0.75%, 1% or 1.5% agar. (A-D) Col-0 (E-H) Ws-4. (A, E) Seedling phenotype, (B, F) primary root projections and overlap (purple), (C, G) HGI values, (D, H) root length. Two-way ANOVA was used to determine the significance between different agar concentrations of Col-0 (HGI (C): *F*_2,319_ = 48.89, *p* < 0.001; root length (D): *F*_2,319_ = 212.4, *p* < 0.001) and Ws-4 (HGI (G): *F_2_*_,325_ = 73.91, *p* < 0.001; root length (H): *F_2_*_,325_ = 46.87, *p* < 0.001); the significant differences between different P_i_ concentrations of Col-0 (HGI (C): *F*_2,319_ = 507.1, *p* < 0.001; root length (D): *F*_2,319_ = 574.8, *p* < 0.001) and Ws-4 (HGI (G): *F*_2,325_ = 1039, *p* < 0.001; root length (H): *F_2_*_,325_ = 69.74, *p* < 0.001). Different letters indicate a statistically significant difference (*p* < 0.05) by Tukey HSD post-hoc analysis among different treatments (n = 29-45). Scale bar = 1 cm.

**Supplementary Figure S10: Effect of taxol on P_i_-dependent skewing of Ws-4 roots.**

Ws-4 seedlings were grown with and without 1 μM Taxol for 7 days at indicated P_i_ concentrations. (A) Phenotype seedlings, (B) HGI values. (C) Stereomicroscopic images of the surface of the roots. (D) Angle of epidermal CFR at root elongation zone. (E) Confocal analysis of root elongation zone after propidium iodide staining (red). The data shown are means ± SEM. Two-way ANOVA was used to determine the significance between different P_i_ treatments of (B) (*F*_2,188_ = 435.5, *p* < 0.0001) and (D) (*F*_2,137_ = 131.1, *p* < 0.0001); the significance between Taxol treatments of (B) (*F*_1,188_ = 263.9, *p* < 0.0001) and (D) (*F*_1,137_ = 79.45, *p* < 0.0001). Different letters indicate significant differences (*p* < 0.05) between treatments. All values represent 2 biological replicates with 12–15 seedlings analysed for each replicate. Scale bars: A) 1 cm; C&E) 100 μm.

**Supplementary Figure S11: Effect of microtubule-destabilization drug, propyzamide on PDS in Col-0 roots.**

Arabidopsis Col-0 seedlings were grown for 7 days with or without 3 μM propyzamide at indicated P_i_ concentrations. (A) Phenotype of seedlings. Scale bar = 1 cm. (B) HGI values of root skewing. (C) Stereomicroscopic images of the surface of the roots. (D) Quantitative analysis of epidermal CFR at the elongation zone. (E) Confocal analysis of root elongation zones of seedlings stained with propidium iodide. The data shown are means ± SEM. Two-way ANOVA was used to determine the significance between different P_i_ treatments of (B) (*F*_2,151_ = 64.32, *p* < 0.001) and (D) (*F*_2,151_ = 45.62, *p* < 0.001); the significant differences between propyzamide treatments of (B) (*F*_1,151_ = 980.5, *p* < 0.001) and (D) (*F*_1,151_ = 248.2, *p* < 0.001). Different letters indicate significant differences (*p* < 0.05) between treatments. All values represent 2 biological replicates with 12–15 seedlings analysed for each replicate. Scale bars: A) 1 cm; C&E) 100 μm.

**Supplementary Figure S12: Effect of propyzamide on PDS in Ws-4 roots.**

Arabidopsis Ws-4 seedlings were grown for 7 days with or without 3 μM propyzamide at indicated P_i_ concentrations. (A) Phenotype of seedlings. Scale bar = 1 cm. (B) HGI values of root skewing. (C) Stereomicroscopic images of the surface of the roots. (D) Quantitative analysis of epidermal CFR at the elongation zone. (E) Confocal analysis of root elongation zones of seedlings stained with propidium iodide. The data shown are means ± SEM. Two-way ANOVA was used to determine the significance between different P_i_ treatments of (B) (*F*_2,136_ = 219.3, *p* < 0.001) and (D) (*F*_2,132_ = 92.37, *p* < 0.0001); the significance between propyzamide treatments of (B) (*F*_1,136_ = 88.18, *p* < 0.001) and (D) (*F*_1,132_ = 34.11, *p* < 0.001). Different letters indicate significant differences (*p* < 0.05) between treatments. All values represent 2 biological replicates with 12–15 seedlings analysed for each replicate. Scale bars: A) 1 cm; C&E) 100 μm.

**Supplementary Figure S13: P_i_ promotes right-handed root growth on horizontal agar plates.**

After 4 days of vertical (70°) growth of Col-0 seedlings on ½MS agar plates containing either 300- or 2500 μM P_i_, plates were placed horizontally to follow the subsequent growth direction of the roots. (A, B) Phenotype of 7-day-old seedlings (4 days vertical, 3 days horizontal growth) at 300 μM P_i_ (A) or 2500 μM P_i_ (B). (C) Quantification of the horizontal root growth direction (CW or CCW). Data shown are means ± SEM. Kruskal-Wallis test was performed to identify significant differences. Different letters indicate significant differences (*p*<0.05) among treatments (n = 34-36). (D) Projection of all primary roots at 300 μM P_i_ after plates were placed horizontal to show there is no left- or right preference. (E) P_i_-induced rightward-root growth and coiling is also promoted in lateral roots. Typical example of 10-day-old seedling (4 days vertical, 6 days horizontal growth) grown at 2500 μM P_i_ is shown with red arrows pointing to the clockwise curling of lateral roots.

**Supplementary Figure S14: Gravitropism response is not affected by P_i_ and PDS response is not depending on gravitropic angle.**

Col-0 seedlings were grown vertically (70°) on ½MS agar plates containing either 300-, 625-, or 1250 μM P_i_. After 4 days, plates were rotated for 90°, either clockwise (CW) or counterclockwise (CCW) or not rotated at all (control). Root tips were recorded every 24 h for 4 additional days and the angles between the vertical gravitropic axis and root axis were determined using ImageJ. (A) Diagram of gravitropic response after 90° plate rotation. (B-D) Quantitative analysis of root gravitropic angles with or without rotation of the agar plate: (B) no rotation, (C) CW-, (D) CCW rotation. Data represents mean ± SEM (n = 27-35). Asterisks indicate a statistically significant difference (**p*<0.05, ***p* < 0.01, ****p*<0.001) between different treatments (300 vs 625 µM P_i_, 1250 vs 625 µM P_i_) by two-way ANOVA. If PDS depended on gravitropism, different effects of the CW and CCW rotations were expected (as PDS is to one direction, i.e. left). In contrast, in both cases, a dose-dependent PDS response was found.

**Supplementary Figure S15: Auxin and Strigolactone appear not to be involved in PDS signalling.**

Col-0 wild type and mutant seedlings of auxin- (*tir1, afb1, and tir1afb1afb3*) or strigolactone (SL) signalling (*max1, max3, max4, d27, max2, d14, cyp706-1,cyp706-2, ubox-1, ubox-2*) were grown for 9 days at three different P_i_ concentrations: 300-, 625-, or 1250 μM (A, B) Primary root projection and overlap (purple) of SL (A) or auxin mutants (B). (C, D) HGI values of SL mutants (C) or auxin mutants (D). Data represents mean ± SEM (n = 14-91). Asterisks indicate a statistically significant difference (**p* < 0.05, ***p* < 0.01, ****p*<0.001) by two-way ANOVA. Scale bar = 1 cm.

**Supplementary Figure S16: Heatmap showing the different expression levels of skewing-related genes in Arabidopsis Col-0 roots at different P_i_ concentrations.**

Heatmap showing expression levels of skewing-related genes (listed in Table S1) in 9-day-old Arabidopsis Col-0 roots grown at three different P_i_ concentrations. Log_2_fold-changes (log_2_FC) between different P_i_ concentrations (i.e. P300 *vs* P625, P625 *vs* P1250 and P300 *vs* P1250) were used as input. Positive log_2_FC mean upregulation when P_i_ levels increased from 300 μM to 625 μM, 625 μM to 1250, or 300 μM to 1250 μM, while negative log_2_FC values indicate downregulation. Significantly different means (**p* < 0.05, ***p* < 0.01, ****p* < 0.001).

**Legends Videos**

**Video S1**: **Time-lapse of root growth and skewing of Col-0 (left) and Ws-4 (right) seedlings at 300 μM P_i_.**

Seedlings were grown for 3 days on ½MS plates containing 300 μM P_i_. Plates were then transferred to a home-made imaging device in the growth chamber (16 h light / 8 h dark) and images were captured every 10 min for 6 days. Pictures in 8 h dark period were invisible and have been removed. ImageJ was used to make the video.

**Video S2**: **Time-lapse of root growth and skewing in Col-0 (left) and Ws-4 (right) at 1250 μM P_i_.**

Seedlings were grown for 3 days on ½MS plates containing 1250 μM P_i_. Plates were then transferred to a home-made imaging device in the growth chamber (16 h light / 8 h dark) and images were captured every 10 min for 6 days. Pictures in 8 h dark period were invisible and have been removed. ImageJ was used to make the video.

**Legends Dataset**

**Dataset S1**: **Source data in this study.**

All the source data in this study are provided and list in Dataset S1.

**References S1**

**Aung K, Lin S-I, Wu C-C, Huang Y-T, Su C, Chiou T-J**. **2006**. pho2, a phosphate overaccumulator, is caused by a nonsense mutation in a MicroRNA399 target gene. *Plant Physiology* **141**: 1000–1011.

**Bisgrove SR, Lee Y-RJ, Liu B, Peters NT, Kropf DL**. **2008**. The microtubule plus-end binding protein EB1 functions in root responses to touch and gravity signals in Arabidopsis. *The Plant Cell* **20**: 396–410.

**Buschmann H, Fabri CO, Hauptmann M, Hutzler P, Laux T, Lloyd CW, Schäffner AR**. **2004**. Helical growth of the Arabidopsis mutant tortifolia1 reveals a plant-specific microtubule-associated protein. *Current Biology* **14**: 1515–1521.

**Bustos R, Castrillo G, Linhares F, Puga MI, Rubio V, Pérez-Pérez J, Solano R, Leyva A, Paz-Ares J**. **2010**. A central regulatory system largely controls transcriptional activation and repression responses to phosphate starvation in Arabidopsis. *PLoS Genetics* **6**: e1001102.

**Furutani I, Watanabe Y, Prieto R, Masukawa M, Suzuki K, Naoi K, Thitamadee S, Shikanai T, Hashimoto T**. **2000**. The SPIRAL genes are required for directional control of cell elongation in Arabidopsis thaliana. *Development* **127**: 4443–4453.

**González E, Solano R, Rubio V, Leyva A, Paz-Ares J**. **2005**. PHOSPHATE TRANSPORTER TRAFFIC FACILITATOR1 is a plant-specific SEC12-related protein that enables the endoplasmic reticulum exit of a high-affinity phosphate transporter in Arabidopsis. *Plant Cell* **17**: 3500–3512.

**Hamburger D, Rezzonico E, MacDonald-Comber Petétot J, Somerville C, Poirier Y**. **2002**. Identification and characterization of the Arabidopsis PHO1 gene involved in phosphate loading to the xylem. *The Plant Cell* **14**: 889–902.

**Hobe M, Müller R, Grünewald M, Brand U, Simon R**. **2003**. Loss of CLE40, a protein functionally equivalent to the stem cell restricting signal CLV3, enhances root waving in Arabidopsis. *Development Genes and Evolution* **213**: 371–381.

**Kato M, Tsuge T, Maeshima M, Aoyama T**. **2019**. Arabidopsis PCaP2 modulates the phosphatidylinositol 4,5-bisphosphate signal on the plasma membrane and attenuates root hair elongation. *The Plant Journal* **99**: 610–625.

**Liu T-Y, Huang T-K, Tseng C-Y, Lai Y-S, Lin S-I, Lin W-Y, Chen J-W, Chiou T-J**. **2012**. PHO2-dependent degradation of PHO1 modulates phosphate homeostasis in Arabidopsis. *The Plant Cell* **24**: 2168–2183.

**Roudier F, Fernandez AG, Fujita M, Himmelspach R, Borner GHH, Schindelman G, Song S, Baskin TI, Dupree P, Wasteneys GO, *et al.*** **2005**. COBRA, an Arabidopsis extracellular glycosyl-phosphatidyl inositol-anchored Protein, specifically controls highly anisotropic Expansion through its involvement in cellulose microfibril orientation. *The Plant Cell* **17**: 1749–1763.

**Roy R, Bassham DC**. **2017**. TNO1, a TGN-localized SNARE-interacting protein, modulates root skewing in Arabidopsis thaliana. *BMC Plant Biology* **17**: 1–12.

**Saffer AM, Carpita NC, Irish VF**. **2017**. Rhamnose-containing cell wall polymers suppress helical plant growth independently of microtubule orientation. *Current Biology* **27**: 2248-2259.e4.

**Sakai T, Honing H van der, Nishioka M, Uehara Y, Takahashi M, Fujisawa N, Saji K, Seki M, Shinozaki K, Jones MA, *et al.*** **2008**. Armadillo repeat-containing kinesins and a NIMA-related kinase are required for epidermal-cell morphogenesis in Arabidopsis. *The Plant Journal* **53**: 157–171.

**Sedbrook JC, Carroll KL, Hung KF, Masson PH, Somerville CR**. **2002**. The Arabidopsis SKU5 gene encodes an extracellular glycosyl phosphatidylinositol-anchored glycoprotein involved in directional root growth. *Plant Cell* **14**: 1635–1648.

**Shih HW, Miller ND, Dai C, Spalding EP, Monshausen GB**. **2014**. The receptor-like kinase FERONIA is required for mechanical signal transduction in Arabidopsis seedlings. *Current Biology* **24**: 1887–1892.

**Van der Does D, Boutrot F, Engelsdorf T, Rhodes J, McKenna JF, Vernhettes S, Koevoets I, Tintor N, Veerabagu M, Miedes E, *et al.*** **2017**. The Arabidopsis leucine-rich repeat receptor kinase MIK2/LRR-KISS connects cell wall integrity sensing, root growth and response to abiotic and biotic stresses. *PLoS Genetics* **13**: e1006832.

**Wang Y, Wang B, Gilroy S, Wassim Chehab E, Braam J**. **2011**. CML24 is involved in root mechanoresponses and cortical microtubule orientation in Arabidopsis. *Journal of Plant Growth Regulation* **30**: 467–479.

**Wang X, Zhu L, Liu B, Wang C, Jin L, Zhao Q, Yuan M**. **2007**. Arabidopsis microtubule-associated protein18 functions in directional cell growth by destabilizing cortical microtubules. *Plant Cell* **19**: 877–889.

**Yang X, Wang B, Farris B, Clark G, Roux SJ**. **2015**. Modulation of root skewing in Arabidopsis by apyrases and extracellular ATP. *Plant and Cell Physiology* **56**: 2197–2206.

**Yao M, Wakamatsu Y, Itoh TJ, Shoji T, Hashimoto T**. **2008**. Arabidopsis SPIRAL2 promotes uninterrupted microtubule growth by suppressing the pause state of microtubule dynamics. *Journal of Cell Science* **121**: 2372–2381.

**Yue X, Guo Z, Shi T, Song L, Cheng Y**. **2019**. Arabidopsis AGC protein kinases IREH1 and IRE3 control root skewing. *Journal of Genetics and Genomics* **46**: 259–267.

**Yuen CYL, Pearlman RS, Silo-suh L, Hilson P, Carroll KL, Masson PH**. **2003**. WVD2 and WDL1modulate helical organ growth and anisotropic cell expansion in Arabidopsis. *Plant Physiology* **131**: 493–506.

**Yuen CYL, Sedbrook JC, Perrin RM, Carroll KL, Masson PH**. **2005**. Loss-of-function mutations of ROOT HAIR DEFECTIVE3 suppress root waving, skewing, and epidermal cell file rotation in Arabidopsis. *Plant Physiology* **138**: 701–714.
